# SAQC: SNP Array Quality Control

**DOI:** 10.1186/1471-2105-12-100

**Published:** 2011-04-18

**Authors:** Hsin-Chou Yang, Hsin-Chi Lin, Meijyh Kang, Chun-Houh Chen, Chien-Wei Lin, Ling-Hui Li, Jer-Yuarn Wu, Yuan-Tsong Chen, Wen-Harn Pan

**Affiliations:** 1 Institute of Statistical Science, Academia Sinica, Taipei 115, Taiwan; 2 Institute of Biomedical Sciences, Academia Sinica, Taipei 115, Taiwan

## Abstract

**Background:**

Genome-wide single-nucleotide polymorphism (SNP) arrays containing hundreds of thousands of SNPs from the human genome have proven useful for studying important human genome questions. Data quality of SNP arrays plays a key role in the accuracy and precision of downstream data analyses. However, good indices for assessing data quality of SNP arrays have not yet been developed.

**Results:**

We developed new quality indices to measure the quality of SNP arrays and/or DNA samples and investigated their statistical properties. The indices quantify a departure of estimated individual-level allele frequencies (AFs) from expected frequencies via standardized distances. The proposed quality indices followed lognormal distributions in several large genomic studies that we empirically evaluated. AF reference data and quality index reference data for different SNP array platforms were established based on samples from various reference populations. Furthermore, a confidence interval method based on the underlying empirical distributions of quality indices was developed to identify poor-quality SNP arrays and/or DNA samples. Analyses of authentic biological data and simulated data show that this new method is sensitive and specific for the detection of poor-quality SNP arrays and/or DNA samples.

**Conclusions:**

This study introduces new quality indices, establishes references for AFs and quality indices, and develops a detection method for poor-quality SNP arrays and/or DNA samples. We have developed a new computer program that utilizes these methods called SNP Array Quality Control (SAQC). SAQC software is written in R and R-GUI and was developed as a user-friendly tool for the visualization and evaluation of data quality of genome-wide SNP arrays. The program is available online (http://www.stat.sinica.edu.tw/hsinchou/genetics/quality/SAQC.htm).

## Background

Single-nucleotide polymorphisms (SNPs), the most abundant genetic markers in the human genome, have been widely used in genetic and genomic research such as studies of disease gene mapping [1-6], medical and clinical diagnostics [7-9], forensic tests [10-12], genome structure of linkage disequilibrium and recombination [13-18], chromosomal aberrations [19-24], and genetic diversity [25-27]. Modern high-throughput and high-resolution SNP array genotyping techniques, such as the Affymetrix GeneChip (Affymetrix Inc., Santa Clara, CA, USA) [28,29] and Illumina BeadChip (Illumina Inc., San Diego, CA, USA) [30-32], provide genotype and fluorescence intensity data on hundreds of thousands of SNPs for each study sample. Many genomic studies are using such SNP genotyping techniques to find marker-trait association via genome-wide association studies [4,6,33] and to identify disease-related chromosomal aberrations via allelic-imbalance analyses [34-39], loss-of-heterozygosity analyses [24,35,40-43], and copy-number analyses [23,24,41,44,45].

Data quality of SNP arrays plays a key role in the accuracy and precision of downstream data analyses. An analysis of contaminated data from poor-quality SNP arrays or genotyping experiments may suggest false-positive and/or false-negative results. Differentiating between reliable and poor-quality SNP arrays is critical to performing downstream statistical data analyses. Quality control of SNP arrays is closely related to a quality assessment of the genotype call of a SNP. Some genotyping algorithms provide SNP-based quality metrics, such as a discrimination signal [46] and confidence scores [47-50]. These metrics mainly focus on a reliability assessment of the genotyping call for individual SNPs rather than an assessment of the overall quality of the SNP arrays. The empirical distributions of most of these metrics were not investigated. Therefore, threshold values for poor quality are often assigned heuristically and not according to a statistical rule. Published reports of systematic analyses to evaluate the data quality of SNP arrays are not available, and good indices that measure the data quality of SNP arrays still await development. Currently, the most broadly used quality measurement of SNP arrays is the genotype call rate (GCR) [51]. GCR, which is the proportion of SNPs whose genotypes can be called on a SNP array, provides a convenient measure for quantification of SNP array quality. GCR is informative and feasible, but this quality metric may be sensitive to the parameters used in genotyping algorithms. For example, "forced call" which leads to a GCR of 100% for a SNP array can always be attained if the least-stringent criterion is used [50].

This study aims to provide a reliable method and related software for the visualization and assessment of the data quality of SNP arrays. We developed new quality indices, derived their empirical distributions, and developed a confidence interval method to identify potentially poor-quality data caused by poor-quality SNP arrays and/or DNA samples. Visualization tools including quality index heatmap plot, quality index polygon plot, AF plot, and genotype call rate plot are integrated into user-friendly software for SNP Array Quality Control (SAQC).

## Methods

### DNA samples and SNP data used in the analyses

Samples used in our analyses were from three genomic projects, the Taiwan Han Chinese Cell and Genome Bank [52], the International HapMap Project [13-16], and the Taiwan Young-Onset Hypertension Study [5]. The first project provides 367 and 448 Han Chinese samples from the Taiwan (TWN) population genotyped using the Affymetrix Human Mapping 100K Set and 500K Set, respectively. Bayesian Robust Linear Model with Mahalanobis Distance Classifier (BRLMM) was used for genotype call analysis [53]. The second project was based on 90 African samples from 30 trios (YRI), 90 European samples from 30 trios (CEU), and 90 independent Asian samples (45 Han Chinese individuals in Beijing [CHB] and 45 Japanese individuals in Tokyo [JPT]). All 270 samples were genotyped using the Affymetrix Human Mapping 100K Set and 500K Set, where Dynamic Model Mapping Analysis [47] and BRLMM were used for genotype call analysis of the Affymetrix Human Mapping 100K Set and 500K Set, respectively. The genotype and hybridization intensity data are publicly available (http://hapmap.ncbi.nlm.nih.gov/). The third project provides 175 and 192 hypertensive patients and 175 and 198 normotensive controls from the TWN population genotyped using the Affymetrix Human Mapping 100K Set and 500K Set, respectively. BRLMM was used for genotype call analysis. We obtained informed consent from all TWN individuals whose samples were used in this study, and this study was approved by the Academia Sinica review board. Based on individual-level AFs in the first two genomic projects, quality indices were calculated for different SNP arrays (Xba and Hind of the Affymetrix 100K Set and Sty and Nsp of the Affymetrix 500K Set) based on samples in various reference populations (the Taiwanese population; ethnic-specific populations; and a combination of African, Asian, and European populations). DNA samples of individuals recruited in the third project were mixed to form four DNA pools with 56, 198, 52 and 192 individuals. Quality indices were calculated for different SNP arrays based on each DNA pool.

### Indices for quantifying SNP array and DNA quality

We introduce the procedures for our new quality index calculations, where individual-level allele frequency (AF) is the key element in the estimation procedures. In contrast to population-level AF which represents a within-population relative frequency of alleles in a population, individual-level AF represents a within-individual relative frequency of alleles in an individual. We measure SNP array quality by quantifying a departure of estimated individual-level AFs from expected AFs via standardized distances. Let {*G*_*n,m*_, *n *= 1,⋯,*N*,*m *= 1,⋯,*M*} denote the genotype and {*λ*_*n,m*_,*n *= 1,⋯,*N*,*m *= 1,⋯,*M*} denote the individual-level AF of the *m*th SNP of the *n*th array in a genotyping experiment of oligonucleotide SNP arrays such as Affymetrix GeneChip (Affymetrix Inc., Santa Clara, CA, USA) and Illumina BeadChip (Illumina Inc., San Diego, CA, USA). Genotypes can be obtained using genotyping calling algorithms [47,49,50,53]. Individual-level AFs can be estimated by calculating adjusted hybridization intensities with the aid of the coefficient of preferential amplification/hybridization (CPA) [54].

To quantify SNP array quality, we first calculated the SNP-level quality index and then calculated the average of the quality indices of the SNPs to obtain an array-level quality index. Two SNP-level quality indices, genotype-based quality index and nearest-mean-based quality index, were developed. Both indices are standardized distances. Where the *m*th SNP with genotype *G*_*m *_is *AA*, *Aa*, or *aa*, the genotype-specific mean and standard deviation of individual-level AFs were calculated as follows:

and *I*[*E*] is an indicator taking a value of 1 if event *E *holds; otherwise, the value is 0.

AF references were established as a collection of genotype-specific mean and standard deviation of individual-level AFs. The genotype and individual-level AF data used to construct AF references can come from samples of the current study or from independent reference samples described in the Results. Genotype-based standardized distance of an individual-level AF was defined as follows:

In some situations, genotype information may be inaccurate. For example, genotypes of SNPs involved in regions of copy number change or chromosomal aberrations may not truly reflect the underlying combination of alleles. Therefore, we developed another index, nearest-mean-based index, without incorporating the genotypes from other genotype calling methods. This property also makes our methods more self-contained. The AF mean and standard deviation of the genotype closest to the observed individual-level AF *λ*_*n,m *_were calculated as follows:

The non-genotype-based (nearest-mean-based) standardized distance of an individual-level AF was calculated as follows:

Next, an array-level quality index was introduced. Let *q*_*x,n *_(*ρ*) denote the *ρ *quantile of genotype-based or nearest-mean-based SNP-level quality indices {*q*_*x,n,m*_,*m *= 1,⋯,*M*} for the *n*th array. To include tolerance for the interference of a small proportion of extreme values that occasionally occurred at some SNPs because of uncontrollable factors, we used a robust statistic, the winsorized mean quality index, to summarize distances of overall SNPs interrogated on a SNP array as follows:

where the top *ρ *of standardized distances was winsorized (i.e., replaced with the observation of the *ρ *quantile) in the calculation. The proposed distance-based quality indices quantify discrepancies between the observed and expected individual-level AFs and tend to have a higher value if the quality of a SNP array is poor. Quality indices based on genotype-based standardized distance and non-genotype-based (nearest-mean-based) standardized distance were defined as *Q*_1 _and *Q*_2_, respectively.

In addition, a confidence interval method was developed to identify poor-quality SNP arrays and/or DNA samples. SNP arrays for which their quality indices exceeded an upper confidence limit based on reference samples were identified as questionable SNP arrays. Quality index references were established as a collection of the upper confidence limits that was obtained by calculating 95%, 97.5%, and 99% quantiles of the underlying empirical distributions of quality indices for different SNP arrays based on samples in various reference populations. Reference populations and empirical distributions of quality indices are described in the Results.

### Performance analysis of quality indices

To evaluate performance of the proposed quality indices, we analyzed authentic data sets and simulated data sets. Details of authentic data sets are presented in the Methods. The simulation procedure was performed as follows. Genomic data from 100 SNP arrays were generated to mimic the real genomic patterns of chromosome 19 of Affymetrix Human Mapping 100K and 500K Sets. The number of SNPs on the chromosome was 690 and 6,396, respectively. The simulation was replicated 1,000 times. The data generation procedure for a SNP was performed as follows. First, at each SNP locus, the number of SNPs with genotypes *AA*, *Aa*, and *aa *on the 100 SNP arrays was generated from a multinomial distribution *MNL*(*N = *100;), where the cell probabilities were population-level genotype frequencies from our real data. Second, the individual-level AF of allele *A *for an individual with genotype *G *for the study SNP was randomly generated from a beta distribution *λ*_*G *_~ *Beta *(*α*_*G*_, *β*_*G*_), where  and  were derived using a moment estimation method, and *μ*_*G *_and *σ*_*G *_denote the sample mean and standard deviation of individual-level AFs. The variance  reflects a total variation (*V*_T,__*G*_) of individual-level AFs in a SNP array, which is the sum of a systematic variation (*V*_s,__*G*_) and an extra variation (*V*_E,__*G*_). The systematic variation reflects the variation of individual-level AFs from samples and arrays with good quality, and the extra variation represents the variation introduced by poor quality of SNP arrays or DNA samples additionally. Let *r *= *V*_E,__*G*_/*V*_T,__*G *_denote the relative extra error for different genotypes; the larger the value, the poorer the SNP array. In other words, AF plot shows broader bands for the larger *r *and, expectedly, a poor sample/array with the larger *r *should be easier to be detected. Third, to mimic practical scenarios, parameters *μ*_*G *_and *V*_s,__*G *_were assigned by empirical means and variances of individual-level AFs from the real data. A relative experimental error of *r *from 0 to 0.6 with increments of 0.025 was considered.  and  were calculated under specified values of , and *r*, and then individual-level AFs were generated from the beta distribution. The 95%, 97.5%, and 99% quantiles of quality index under *r *= 0 were derived to serve as an upper confidence limit for identification of poor-quality SNP arrays. For each relative experimental error *r*, a proportion of SNP arrays that were identified as poor-quality SNP arrays was calculated in each simulation replication. An average and a standard deviation of proportions of poor-quality SNP arrays in 1,000 simulations were calculated.

## Results

### Empirical distributions and upper confidence limits of quality indices

We calculated quality indices and established their empirical distributions based on SNP array data from the Taiwan Han Chinese Cell and Genome Bank [52] and the International HapMap Project [13-16]. Values of quality indices were fitted by lognormal distributions and examined by Kolmogorov-Smirnov goodness-of-fit tests [55]. P-values of all goodness-of-fit tests were >0.05 for SNP arrays and study populations, demonstrating that the quality index was well modeled by lognormal distributions (Additional file 1).

**Figure 1:**
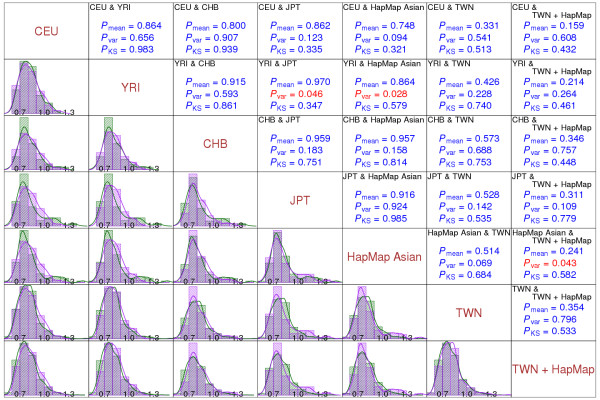
**Comparison of the distributions of quality indices from the different ethnic groups based on the Affymetrix Human Mapping 500K Set**. The subfigures in the diagonal show the names of ethnic groups (CEU, YRI, CHB, JPT, HapMap Asian, TWN, and TWN + HapMap). The subfigures in the lower diagonal part show the histograms and fitted curves of quality index *Q*_2 _for any two given ethnic groups. In each subfigure, distributions of quality indices from two ethnic groups are superimposed, with the purple bar and curve corresponding to the ethnic group named on the above, and the green bar and curve corresponding to the ethnic group named on the right. The subfigures in the upper diagonal part show the p-values for testing equality of means (in log scale), variances (in log scale) and sampling distributions for any two given ethnic groups, *P*_mean_, *P*_var _and *P*_KS_. The names of the two ethnic groups compared are shown in the upper-left corner in each subfigure. P-values smaller than 0.05 are indicated in red.

We compared quality indices among different ethnic groups. In addition to a pairwise comparison of histograms for quality indices from different ethnic groups, we also formally compared the distributions of quality indices from different ethnic groups by testing the equalities of their means (in log scale), variances (in log scale) and sampling distributions using two-sample Z test, F test and Kolmogorov-Smirnov goodness-of-fit test, respectively. We analyzed SNPs interrogated on the Affymetrix 500K Set with all chromosomes combined. The results showed that, with very few exceptions (highlighted in red), there were no significant differences in means, variances and distributions of quality indices across ethnic groups in general (Figure 1).

We also evaluated the effect of laboratory on quality indices by controlling the population effect. We compared distributions of quality indices for the samples from two closely-related ethnic groups. The first group was Han Chinese residing in Taiwan and referred as TWN samples in this study (n = 448), and the second group was Han Chinese residing in Beijing and referred as CHB samples in the International HapMap Project (n = 45). These two groups of samples were genotyped in different laboratories. Kolmogorov-Smirnov goodness-of-fit test was employed to test the equality of quality indices for the two distributions. The p-value was 0.573, which suggested that genotyping done in different laboratories did not have a significant effect of the distribution of quality indices. The 95%, 97.5%, and 99% upper confidence limits of quality indices for different SNP arrays based on samples in various reference populations including the Taiwanese, African, Asian, and European populations (i.e., population-specific confidence limits) and the samples in all reference populations (i.e., combined-population confidence limits) were calculated and then provided in SAQC software. The confidence limits provided thresholds for identifying poor-quality SNP arrays and/or DNA samples using the proposed confidence interval method.

### Quality evaluation of real SNP arrays and DNA samples

**Figure 2:**
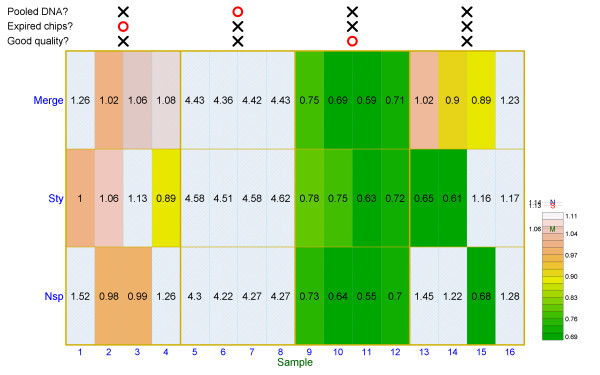
**Quality index heatmap plot for SNP arrays of different quality and pooled DNA**. This figure shows quality index *Q*_2 _for Nsp arrays, Sty arrays, and merged Nsp and Sty arrays for 16 DNA samples. The first four samples and the second four samples were genotyped using unexpired arrays. The third four samples were genotyped with expired arrays. The last four samples were DNA pools constructed by 56, 198, 52 and 192 individuals and genotyped using unexpired arrays, respectively. The magnitude of the quality index is presented using a heatmap plot for which a color spectrum from dark green to white represents the magnitude range. The levels of the quality index (*μ*, *μ *+ *σ*, *μ *+ 2*σ*, *μ *+ 3*σ*, *μ *+ 4*σ*, *μ *+ 5*σ *and *μ *+ 6*σ*) are given, where *μ *and *σ *denote the sample mean and standard deviation, respectively, of the quality index for the normal reference samples. Moreover, the upper limits of the quality index for arrays Nsp (N), Sty (S), and Merge (M) are also given in the key. For the 16 DNA samples, if the values of the quality indices of arrays Nsp, Sty and Merge are greater than these upper limits, arrays are presented in light-blue shaded views, respectively.

Eight experimentally designed bad-quality SNP assays were used to validate our new quality index calculations (Samples 1 - 8 in Figure 2). Samples 1 - 4 were individual DNA with good quality from the Taiwan Han Chinese Cell and Genome Bank [52] and genotyped using arrays beyond expiration date (expired arrays); Samples 5 - 8 were pooled DNA of multiple individuals from the Taiwan Young-Onset Hypertension Study [5] and genotyped using arrays prior to expiration date (unexpired arrays). All the eight samples were genotyped with the Affymetrix Human Mapping 500K Set (Nsp and Sty arrays), and the quality index *Q*_2 _was calculated for the Nsp array and Sty array and the "Merge" array which contains all SNPs on the Nsp array and Sty array. For the TWN population, the 95%, 97.5%, and 99% quantiles of the quality index in the reference samples are, respectively, 1.144, 1.246, and 1.385 for Nsp arrays; 1.133, 1.233, and 1.367 for Sty arrays; and 1.056, 1.129, and 1.224 for Merge arrays. A SNP array with a low quality index (good quality) is presented in green, and a SNP array with a high quality index (poor quality) is presented in white in the quality index heatmap plot. As shown in Figure 2, when the 95% quantile was applied, Samples 1 - 8 showed poor performance for both SNP arrays and were categorized as "poor quality". The performance of Samples 5 - 8 was worse than that of Samples 1 - 4. The same analysis method was applied to 448 unselected individuals, which were recruited by the Taiwan Han Chinese Cell and Genome Bank [52] and genotyped using unexpired arrays. The majority of the samples had low quality indices for both SNP arrays and was categorized as "good quality"; four representative samples (Samples 9 - 12) were shown in Figure 2 for illustration. Only few samples had high quality index for at least one SNP array and were categorized as "poor quality"; four of them (Samples 13 - 16) were shown in Figure 2 for illustration.

Furthermore, we picked up the first sample in each category, i.e., Samples 1, 5, 9 and 13, for exemplifying the problems that could be identified by our method. The four samples were further examined using AF plots (Additional file 2). Deviation from a typical AF profile (i.e., three AF bands) was observed in poor-quality SNP arrays with high quality indices. Sample 1 was genotyped using expired arrays and, as expected, showed high quality indices in Nsp and/or Sty array (QI_Nsp _= 1.521, QI_Sty _= 1.001, and QI_Merge _= 1.259) (Additional file 2, Supplemental Figure S2 (A1) and (A2)). A SNP array assay with a set of bad-quality arrays would behave like this. Sample 5 was derived from a DNA pool of 56 TWN individuals with hypertension, and the AF of a SNP reflected population-level AF. As expected, the AFs of this sample were deviated from the upper- and lower-bound of individual-level AFs across the genome, which resulted in extremely high quality indices (QI_Nsp _= 4.305, QI_Sty _= 4.577, and QI_Merge _= 4.433) and thus very poor quality (Additional file 2, Supplemental Figure S2 (B1) and (B2)). Samples with server DNA contamination would show similar AF profiles like that in this subgroup. Sample 9 had low quality indices for the Nsp, Sty, and Merge arrays (QI_Nsp _= 0.733, QI_Sty _= 0.776, and QI_Merge _= 0.753), signifying an accurate hybridization, thereby suggesting good quality of both the DNA sample and SNP arrays. This was typically observed for individual genotyping experiment in this study (Additional file 2, Supplemental Figure S2 (C1) and (C2)). Sample 13 showed poor quality in the Nsp array but good quality in the Sty array (QI_Nsp _= 1.446, QI_Sty _= 0.647, and QI_Merge _= 1.024), indicating that the unsatisfactory quality of this sample was caused by the Nsp array assay or genotyping error rather than the original DNA sample (Additional file 2, Supplemental Figure S2 (D1) and (D2)). If the error was caused by poor-quality DNA, inadequate performance should have been found in both the Nsp and Sty arrays.

### Results of simulation studies

**Figure 3:**
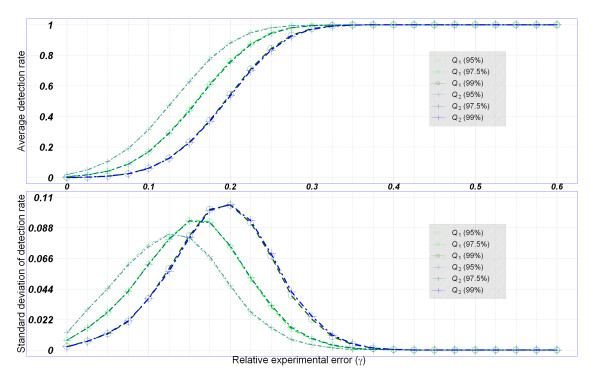
**Detection rate of quality indices in the simulation study based on the Affymetrix 100K SNP arrays**. Averages and standard deviations of detection rates of the genotype-based and nearest-mean-based quality indices {*Q*_1_(*ρ*), *Q*_2_(*ρ*), *ρ *= 95%, 97.5%, 99%} for a relative experimental error *r *of 0-60%. The data were generated from the TWN population.

**Figure 4:**
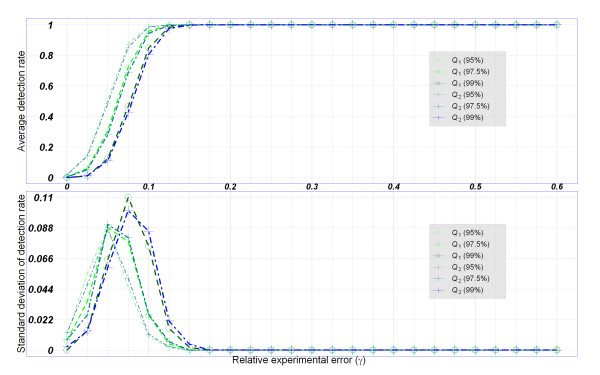
**Detection rate of quality indices in the simulation study based on the Affymetrix 500K SNP arrays**. Averages and standard deviations of detection rates of the genotype-based and nearest-mean-based quality indices {*Q*_1_(*ρ*), *Q*_2_(*ρ*), *ρ *= 95%, 97.5%, 99%} for a relative experimental error *r *of 0-60%. The data were generated from the TWN population.

We defined detection rate as a proportion of poor-quality SNP arrays detected by the proposed confidence interval method according to a 95%, 97.5%, or 99% quantile of quality index. We calculated the mean and standard deviation of detection rates of 1,000 simulations at a relative experimental error (*r*) of 0-0.6 at increments 0.025. Results of the Affymetrix 100K and Affymetrix 500K Sets based on the TWN population are shown in Figure 3 and Figure 4.

First, the effect of the relative experimental error (*r*) is discussed. The false detection rates (i.e., detection rate at *r *= 0) were small, and true detection rates (i.e., detection rate at *r *> 0) increased as the relative experimental error *r *increased. The average detection rates followed S-shaped curves when plotted average detection rate versus *r *(Figure 3 and Figure 4). The precision of detection rates was assessed using the standard deviation of detection rates (Figure 3 and Figure 4). Second, the performance of two quality indices (*Q*_1 _and *Q*_2_) was compared. We found that the two indices have similar detection rates and precision in our simulation (Figure 3 and Figure 4). The impact of ethnic populations (TWN, CHB + JPT, and Combined) on the average detection rates and precision of detection rates was evaluated. The patterns of detection rates were quite similar in different ethnic populations although the simulation data were generated from genomic distributions of various populations (Additional file 3, Figure 3 and Figure 4). Fourth, the impact of the SNP genotyping platform (Affymetrix 100K and 500K Sets) was also assessed. In general, the Affymetrix 500K Set with a higher marker density (Figure 4) had a higher detection rate than the Affymetrix 100K Set (Figure 3). For the Affymetrix 100K Set, almost 100% of poor-quality SNP arrays were identified successfully when *r *was >0.35; and for the Affymetrix 500K Set, almost 100% of poor-quality SNP arrays were identified successfully when *r *was >0.15 (Figure 3 and Figure 4).

Fifth, the impact of winsorization thresholds (*ρ*) was also evaluated. In general, average detection rates presented similar S-shaped curves, whereas standard deviations of detection rates presented similar unimodal curves (Figure 3 and Figure 4). Quality indices with a lower winsorization threshold had higher true detection rates at *r *> 0 but were penalized by a slightly higher false detection rate and standard deviation of detection rate at *r *= 0 (Figure 3 and Figure 4).

### SAQC software

**Figure 5:**
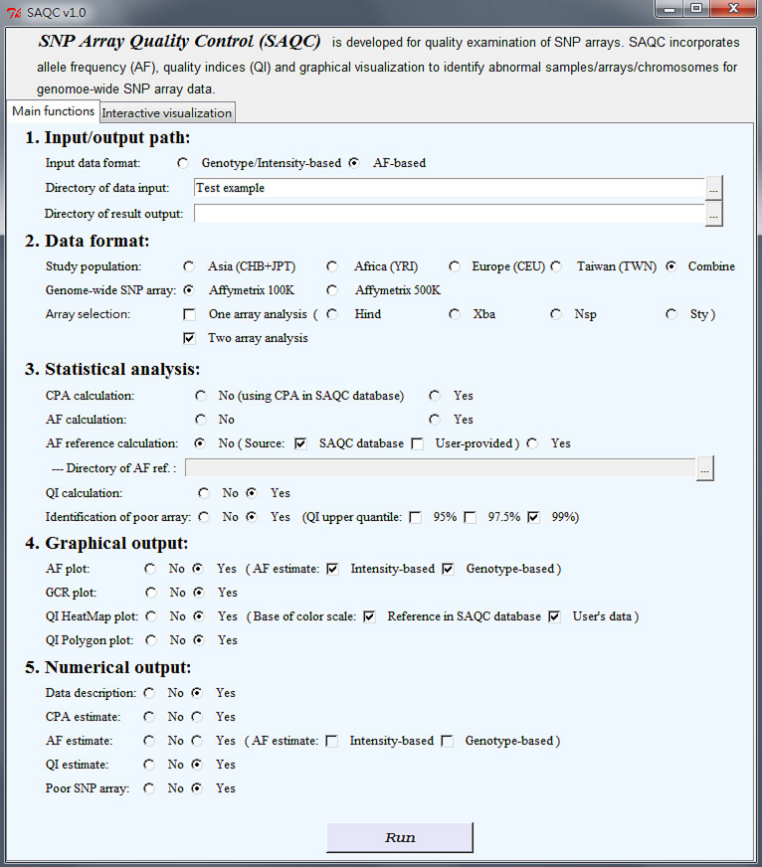
**Interface of main functions of new SAQC software to assess data quality of SNP arrays**. The main functions of SAQC software contain five items: input/output path, data format, statistical analysis, graphical output and numerical output.

**Figure 6:**
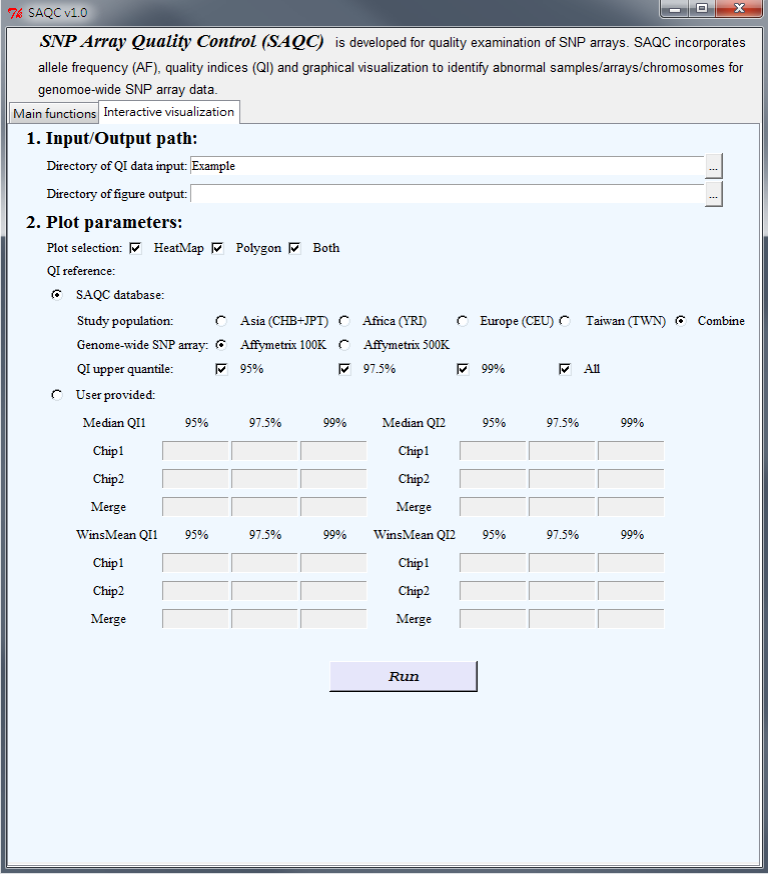
**Interface of interactive visualization of new SAQC software to assess data quality of SNP arrays**. The interactive visualization contains two items: input/output path and plot parameters.

SAQC software with R-GUI interfaces (Figure 5 and Figure 6) is available online (http://www.stat.sinica.edu.tw/hsinchou/genetics/quality/SAQC.htm). The test examples are also provided, and the examples can be run conveniently by simply clicking the button "Run" once SAQC software has been initialized. SAQC software consists of two components: (1) main functions (Figure 5), and (2) interactive visualization (Figure 6). The main functions provide statistical analyses of genotype and hybridization intensity data or AF data and produce both graphical and numerical results of quality indices. The interactive visualization provides an interactive mode to display the results of quality indices. The functions are illustrated in detail as follows:

#### Component 1 - Main functions

(1) Input/output path: Users select the input data format, where either genotypes and hybridization intensity data or AF data can be selected. Data will be automatically loaded by searching data files in the specified input directory. Numerical outputs and graphical outputs will be saved in the specified output directory.

(2) Data format: We provide CPA, AF, and QI reference databases for HapMap Asian (CHB + JPT), African (YRI), European (CEU), Taiwanese (TWN), and combined populations (TWN + CHB + JPT + YRI + CEU). Databases for the Affymetrix 100K/500K are provided, and databases for the Affymetrix Array 6.0 and Illumina 550K BeadChip are being constructed. Users can decide to analyze one array (e.g., Xba or Hind array of the Affymetrix 100K Set) or two arrays (e.g., both Xba and Hind array of the Affymetrix 100K Set).

(3) Statistical analysis: SAQC software provides utilities including CPA calculation, AF estimation, AF reference calculation, QI calculation, and identification of poor-quality arrays. Users can select to construct their own CPA and AF references or to use the references provided by the SAQC databases. In addition, users can also select 95%, 97.5%, or 99% for the upper quantile of the quality index when identifying poor-quality arrays.

(4) Graphical output: SAQC software provides different types of plots including intensity-based and genotype-based AF plots, QI heatmap plots, QI polygon plots, and GCR plots.

(5) Numerical output: SAQC software provides the following numerical outputs including: data description, CPA estimate, AF estimate, QI estimate, and poor-quality SNP array. In addition, a file that shows a sample list and GCR for each SNP array, and a log file that shows the progress of program execution and error/warning messages are included.

#### Component 2 - Interactive visualization

(1) Input/output path: Users can specify the input and output directories. Quality index data will be automatically loaded by searching data files in the specified input directory. Graphical outputs will be saved in the specified output directory.

(2) Plot parameters: Users first select to display a QI heatmap plot (as in Additional file 4, Supplemental Figure S4 (A)) and/or QI polygon plot (as in Additional file 4, Supplemental Figure S4 (B)) and then choose suitable graphic settings for the plots. Users can either apply the parameters established from the SAQC databases of different ethnic populations and SNP array platforms, or they can also provide their own references.

## Discussion

The sampling distribution of quality indices is important to systematically identify poor-quality SNPs and SNP arrays. Although other quality indices for single SNPs have been proposed [46-50], their sampling distributions were seldom investigated. In this report, we proposed new quality indices and tested them. We derived sampling distributions for the quality indices through empirical studies of several large genomic projects. We found that the proposed quality indices follow lognormal distributions. A similar conclusion was also reached in our simulation study. For example, for *Q*_2_, only a small proportion, 2.2% for the Affymetrix 100K Set and 4.0% for the Affymetrix 500K Set, of the Kolmogorov-Smirnov goodness-of-fit tests rejected the null hypothesis "quality indices follow lognormal distributions" (P-value < 0.05) at a relative experimental error *r *= 0, meaning that the proposed quality indices can be well modeled by lognormal distributions.

The proposed quality indices were compared with other indices. In addition to the winsorized mean, other robust metrics, such as the median and trimmed mean, can be used to calculate an array-level quality index. We thus compared the performance of the winsorized mean and median in simulation studies (compare Additional file 3 with Additional file 5). The results showed that the median statistic also was effective at evaluating SNP array quality. The winsorized mean statistic did, however, have a consistently higher true detection rate than did the median statistic, especially when *r *was <0.4 for the Affymetrix 100K Set and <0.175 for the Affymetrix 500K Set (compare Additional file 3 with Additional file 5). Moreover, we also compared the proposed quality indices with the commonly used index GCR. In general, SNP arrays with a low GCR often have poor quality indices. For example, for the Affymetrix 500K Set in our study, absolute-value correlation coefficients for the quality index *Q*_2 _and GCR in the merged Nsp and Sty arrays were 0.8264, 0.6732, 0.7951, and 0.8727 for the YRI, CEU, CHB, and JPT populations, respectively (data not shown). Nevertheless, the proposed indices can work in concert with plots of AFs and quality indices to provide complementary information for a GCR index to identify poor-quality SNP arrays and/or DNA samples that cannot be detected by GCR.

A confounding factor, chromosome aneuploidy, should be considered when drawing conclusions from an analysis of the proposed quality indices. A high value for the quality index may be caused by a poor-quality SNP array (true positive) or may be a reflection of DNA samples with chromosomal aneuploidy (false positive). An artifactual high-quality index may result from chromosomal aberrations of the test samples that deviated from the normal references that were used to establish SAQC databases. In fact, changes in the chromosomal structure of DNA samples can be indicative of important biological processes rather than of poor-quality SNP arrays with high experimental noise. One simulated example of a poor-quality SNP array is the triploid cancer patient with high quality indices of Hind, Xba and merged arrays (QI_Hind _= 4.393, QI_Xba _= 7.541, and QI_Merge _= 5.922) (Additional file 6), where *ρ *= 95% was considered. SAQC software overcomes this potential confounding issue by providing intensity-based AF plots. High quality indices that are due to polyploidy or aneuploidy can be easily identified via an intensity-based AF plot (Additional file 6). In addition, SAQC software can be used jointly with our recently developed analysis tool, ALOHA software [39], to identify regions of chromosomal aberrations, such as allelic imbalance, loss of heterozygosity and copy number changes. Although our quality index is not designed for directly detecting copy number alterations, it can be used to select the best ones (i.e., samples with good quality indices) from a set of samples to be used as references to compute absolute copy numbers.

For ethnic populations and laboratory effect, our analyses suggest that the effects of the ethnic population and laboratory are not significant (see Figure 1). Thus, the results will only be changed mildly for wrongly assigned population. SAQC software provides population-specific and combined-population databases of AFs and quality indices for identifying poor-quality SNP arrays and/or DNA samples. Use of the reference from the same population as the study group is recommended. If the desired population is not available in SAQC, users can use the reference from the combined population; alternatively, users can build or provide the references for their own population and their own laboratory using SAQC (see the **SAQC software **section).

The analysis of Sample 5 in Figure 2 illustrates that it is possible to use our proposed method to discern the origin of a bad hybridization signals is the DNA sample or the array for samples. However, the conclusion solely relies on the discordance between the two arrays for the same sample (e.g., Xba and Hind of the Affymetrix 100K Set and Sty and Nsp of the Affymetrix 500K Set), so this application can not be applied to the case of a single array system (e.g., Affymetrix Array 6.0 or Illumina 550K) if no experimental replicates available.

In addition to the Affymetrix Human Mapping 100K and 500K Sets, the new SAQC software can be extended to handle SNP arrays with a higher marker density. Currently, we are establishing CPA, AF, and QI reference databases for the Affymetrix Array 6.0 and Illumina 550K BeadChip. Completion of this task will further enhance the applications of our methods and SAQC software.

## Conclusions

Quality control of SNP arrays plays an important role in downstream data analyses. As a result of our analysis, we have proposed new quality indices and have established their empirical distributions for different SNP array platforms and ethnic populations. We have also developed a detector to assist in identifying poor-quality SNP arrays and/or DNA samples based on empirical distributions of quality indices; this method has been evaluated by analyses of authentic data and simulated data. In addition, the newly developed SAQC software provides an easy-to-use analysis platform for SNP array quality control. In conclusion, an integrated analysis of quality indices (the quality index heatmap plot and quality index polygon plot), AF data (intensity-based AF plot and genotype-based AF plot), and GCR data (GCR plot) is helpful for determining the quality of genome-wide SNP arrays and thereby enhances the reliability of this sophisticated data analysis.

## Availability and requirements

The SAQC software and test examples can be downloaded from the SAQC website: http://www.stat.sinica.edu.tw/hsinchou/genetics/quality/SAQC.htm.

**Project name**: SNP array quality control project

**Project home page**: http://www.stat.sinica.edu.tw/hsinchou/genetics/quality/SAQC.htm

**Operating system**: MS Windows^®^

**Programming language**: Language R and R-GUI

**Other requirements**: No

**Any restrictions to use by non-academics**: On request and citation

## List of abbreviations used

AF: allele frequency; BRLMM: Bayesian Robust Linear Model with Mahalanobis Distance Classifier; CEU: CEPH Utah residents; CHB: Han Chinese in Beijing; CPA: coefficient of preferential amplification/hybridization; GCR: genotype call rate; JPT: Japanese in Tokyo; QI: quality index; SAQC: SNP Array Quality Control; SNP: single-nucleotide polymorphism; TWN: Han Chinese in Taiwan; YRI: Yoruba in Ibadan.

## Authors' contributions

HCY conceived the study, developed statistical methods, and prepared the manuscript. HCL and CWL developed the SAQC program and analyzed the data with HCY. MK constructed DNA pools of the TWN samples. CHC and LHL contributed to discussion and prepared the revision with HCY. JYW, YTC, and WHP provided DNA samples and genotyping support. All authors read and approved the final manuscript.

## Supplementary Material

Additional file 1 **Figure S1--Lognormal distribution of quality index based on the Affymetrix Human Mapping 100K and 500K Sets**. Kolmogorov-Smirnov goodness-of-fit tests were used to examine lognormal distributions of the quality index *Q*_2 _for all study samples. Here, each figure consists of 24 panels. The first 23 panels show a distribution of the quality index for each chromosome, and the twenty-fourth panel presents a whole-genome distribution. In each panel, a histogram (gray bar), theoretical lognormal curve (purple line), and fitted curve (green line) for the quality index are shown, and the number shown in parentheses is the P-value of the Kolmogorov-Smirnov goodness-of-fit test. Three red dashed reference lines show the 95%, 97.5%, and 99% quantile. Samples with aneuploidy, amplification, or very long contiguous homozygous stretches were removed. For the Affymetrix Human Mapping 100K Set, we have (A1) 57 CEU founders, (A2) 58 YRI founders, (A3) 43 CHB samples, (A4) 43 JPT samples, (A5) 86 HapMap Asian samples (43 CHB and 43 JPT), (A6) 360 TWN samples, and (A7) 561 study samples (360 TWN samples and 201 HapMap samples). For the Affymetrix Human Mapping 500K Set, we have (B1) 55 CEU founders, (B2) 59 YRI founders, (B3) 43 CHB samples, (B4) 44 JPT samples, (B5) 87 HapMap Asian samples (43 CHB and 44 JPT), (B6) 442 TWN samples, and (B7) 643 study samples (442 TWN samples and 201 HapMap samples). Click here for file

Additional file 2 **Figure S2--Individual-level AF plots of four samples based on the Affymetrix Human Mapping 500K Set**. AF plots of four samples: (A1) and (A2) are results of Nsp and Sty arrays for sample SC100011 (Sample 1); (B1) and (B2) are results of Nsp and Sty arrays for sample SC100854 (Sample 5); (C1) and (C2) are results of Nsp and Sty arrays for sample SC100444 (Sample 9) genotyped with expired SNP arrays; and (D1) and (D2) are results of Nsp and Sty arrays for pooled DNA samples (Sample 13). The panels display AFs for each of the 23 chromosomes. The horizontal axis is the physical position (unit = 1 Mb), and the vertical axis is the AF. Each SNP is denoted by a blue point, and the gap in each subplot represents the centromeric gap. The distribution of AFs was estimated using a smoothed density function and is shown as a pink curve. Click here for file

Additional file 3 **Figure S3--Detection rates of winsorized mean-based quality indices in the simulation study**. Averages and standard deviations of detection rates of the genotype-based index (*Q*_1_) and nearest-mean-based quality index (*Q*_2_) {*Q*_1_(*ρ*), *Q*_2_(*ρ*), *ρ *= 95%, 97.5%, 99%} for a relative experimental error *r *of 0-60% with increments of 0.025. (A) HapMap Asian (CHB + JPT) population and Affymetrix 100K SNP array. (B) HapMap Asian (CHB + JPT) population and Affymetrix 500K SNP array. (C) The combined population (TWN + CHB + JPT + YRI + CEU) and Affymetrix 100K SNP array. (D) The combined population (TWN + CHB + JPT + YRI + CEU) and Affymetrix 500K SNP array. Click here for file

Additional file 4 **Figure S4--Two interactive plots provided by SAQC software**. (A) Interactive QI heatmap plot. (B) Interactive QI polygon plot. Click here for file

Additional file 5 **Figure S5--Detection rates of median-based quality indices in the simulation study**. Averages and standard deviations of detection rates of the genotype-based index (*Q*_1_) and nearest-mean-based quality index (*Q*_2_) {*Q*_1_(*ρ*), *Q*_2_(*ρ*), *ρ *= 95%, 97.5%, 99%} for a relative experimental error *r *of 0-60% with increments of 0.025. (A) HapMap Asian (CHB + JPT) population and Affymetrix 100K SNP array. (B) HapMap Asian (CHB + JPT) population and Affymetrix 500K SNP array. (C) The combined population (TWN + CHB + JPT + YRI + CEU) and Affymetrix 100K SNP array. (D) The combined population (TWN + CHB + JPT + YRI + CEU) and Affymetrix 500K SNP array. Click here for file

Additional file 6 **Figure S6--Individual-level AF plot of a triploid cancer patient**. Individual-level AF data of a cancer patient were generated by a simulation procedure and then displayed in an AF plot. The panels display AFs for each of the 23 chromosomes. The horizontal axis indicates the physical position (unit = 1 Mb), and the vertical axis shows the AF. Each SNP is denoted by a blue point, and the gap in each subplot represents the centromeric gap. The distribution of AFs was estimated using a smoothed density function and is shown as a pink curve. Click here for file
