# Supplementary material for: SAQC: SNP Array Quality Control
Source: BMC Bioinformatics. 2011 Apr 18;12:100. doi: 10.1186/1471-2105-12-100 (PMC3101186; doi:10.1186/1471-2105-12-100)

**Additional materials**

**Figure S1.**—**Lognormal distribution of quality index based on the Affymetrix Human Mapping 100K and 500K Sets.** Kolmogorov-Smirnov goodness-of-fit tests were used to examine lognormal distributions of the quality index *Q*_2_ for all study samples. Here, each figure consists of 24 panels. The first 23 panels show a distribution of the quality index for each chromosome, and the twenty-fourth panel presents a whole-genome distribution. In each panel, a histogram (gray bar), theoretical lognormal curve (purple line), and fitted curve (green line) for the quality index are shown, and the number shown in parentheses is the P-value of the Kolmogorov-Smirnov goodness-of-fit test. Three red dashed reference lines show the 95%, 97.5%, and 99% quantile. Samples with aneuploidy, amplification, or very long contiguous homozygous stretches were removed. For the Affymetrix Human Mapping 100K Set, we have (A1) 57 CEU founders, (A2) 58 YRI founders, (A3) 43 CHB samples, (A4) 43 JPT samples, (A5) 86 HapMap Asian samples (43 CHB and 43 JPT), (A6) 360 TWN samples, and (A7) 561 study samples (360 TWN samples and 201 HapMap samples). For the Affymetrix Human Mapping 500K Set, we have (B1) 55 CEU founders, (B2) 59 YRI founders, (B3) 43 CHB samples, (B4) 44 JPT samples, (B5) 87 HapMap Asian samples (43 CHB and 44 JPT), (B6) 442 TWN samples, and (B7) 643 study samples (442 TWN samples and 201 HapMap samples).

(A1)
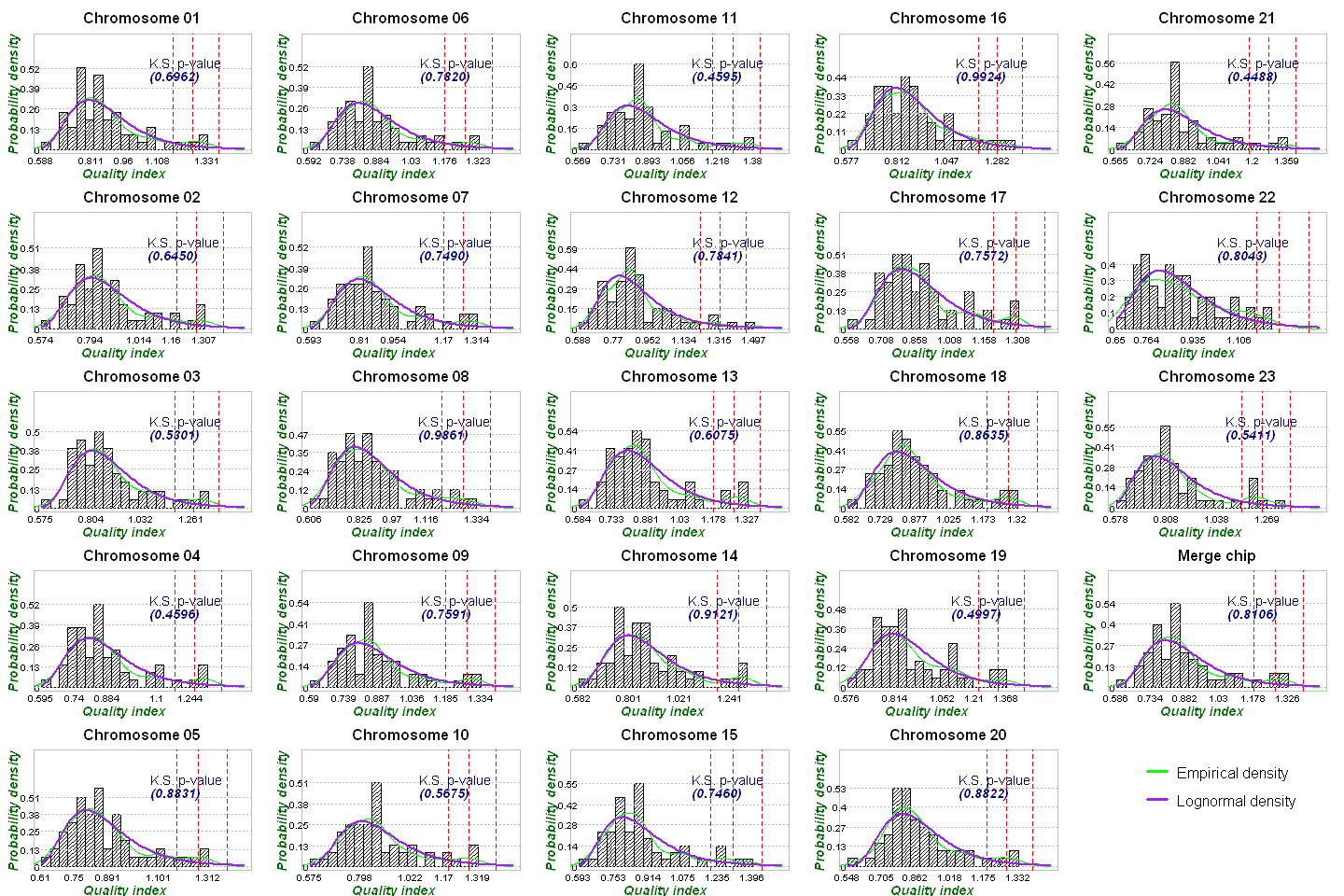


(A2)


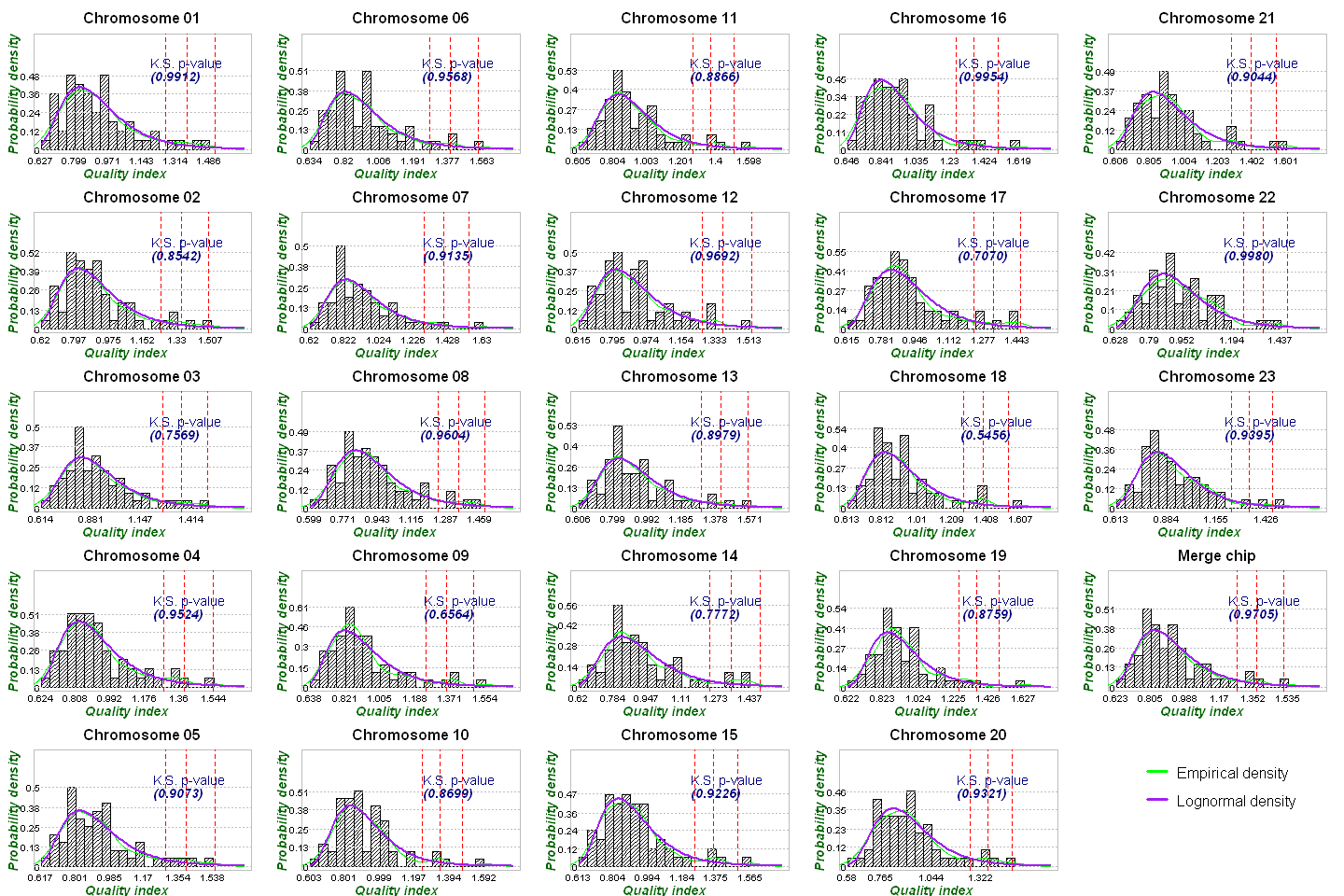


(A3)


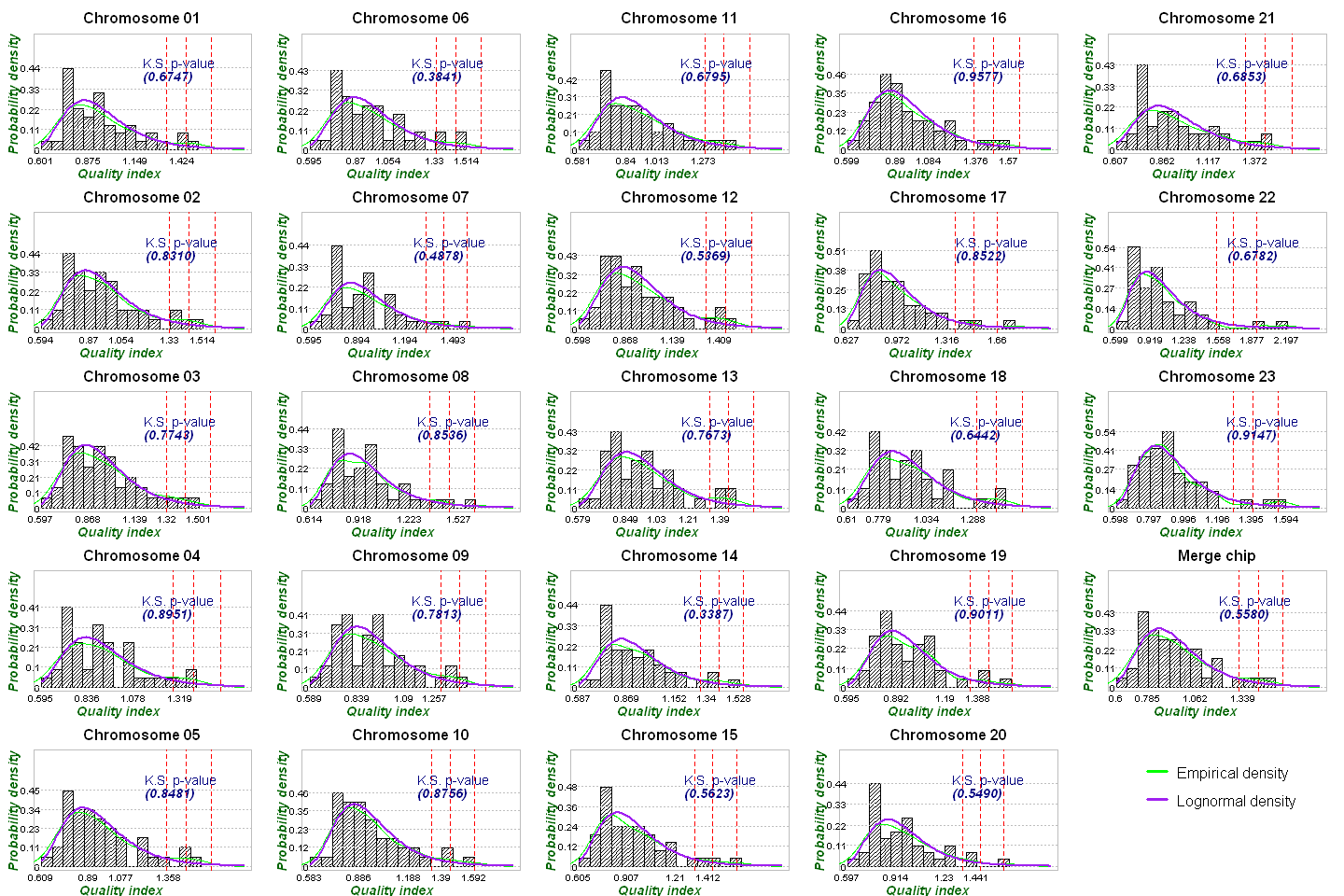


(A4)


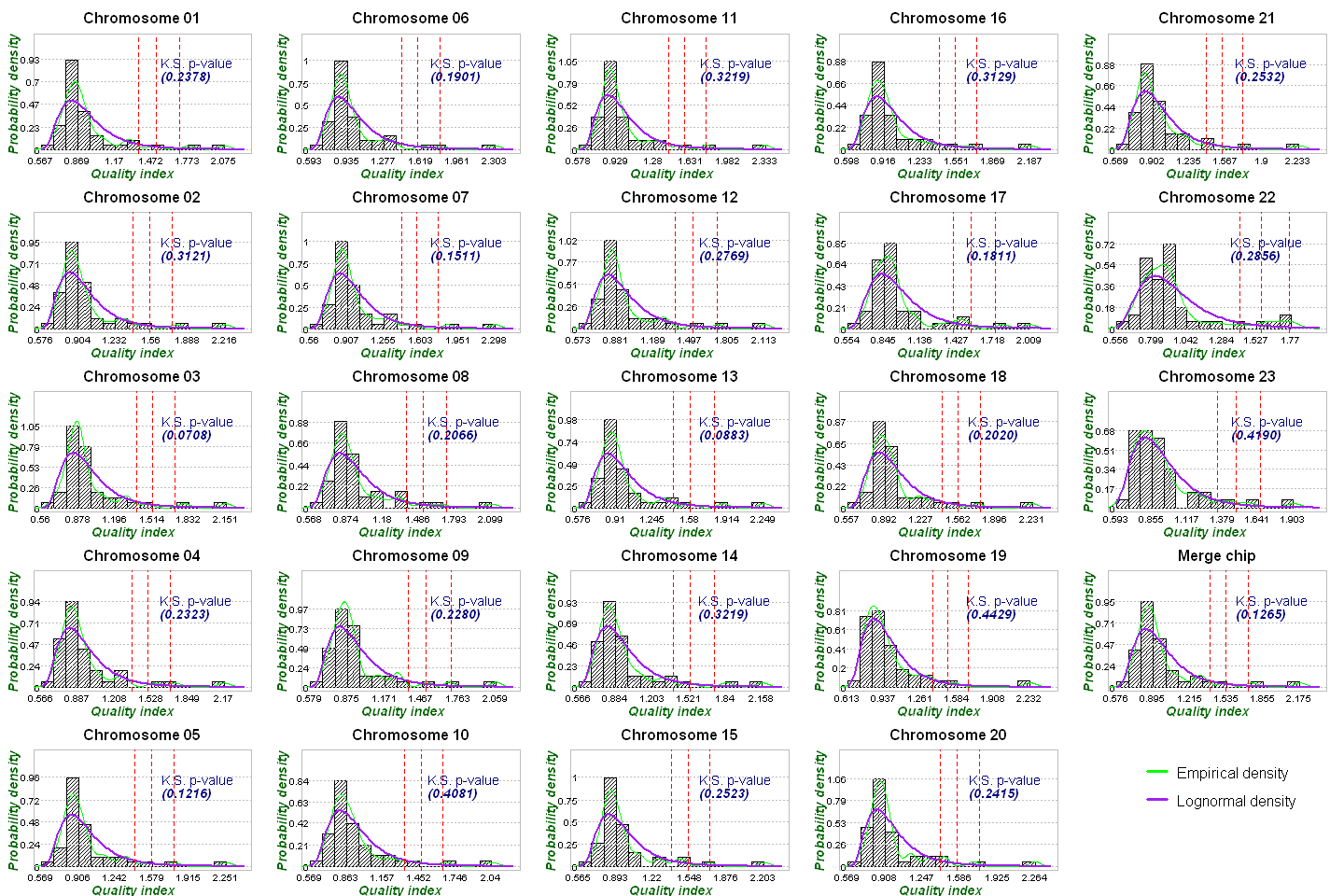


(A5)


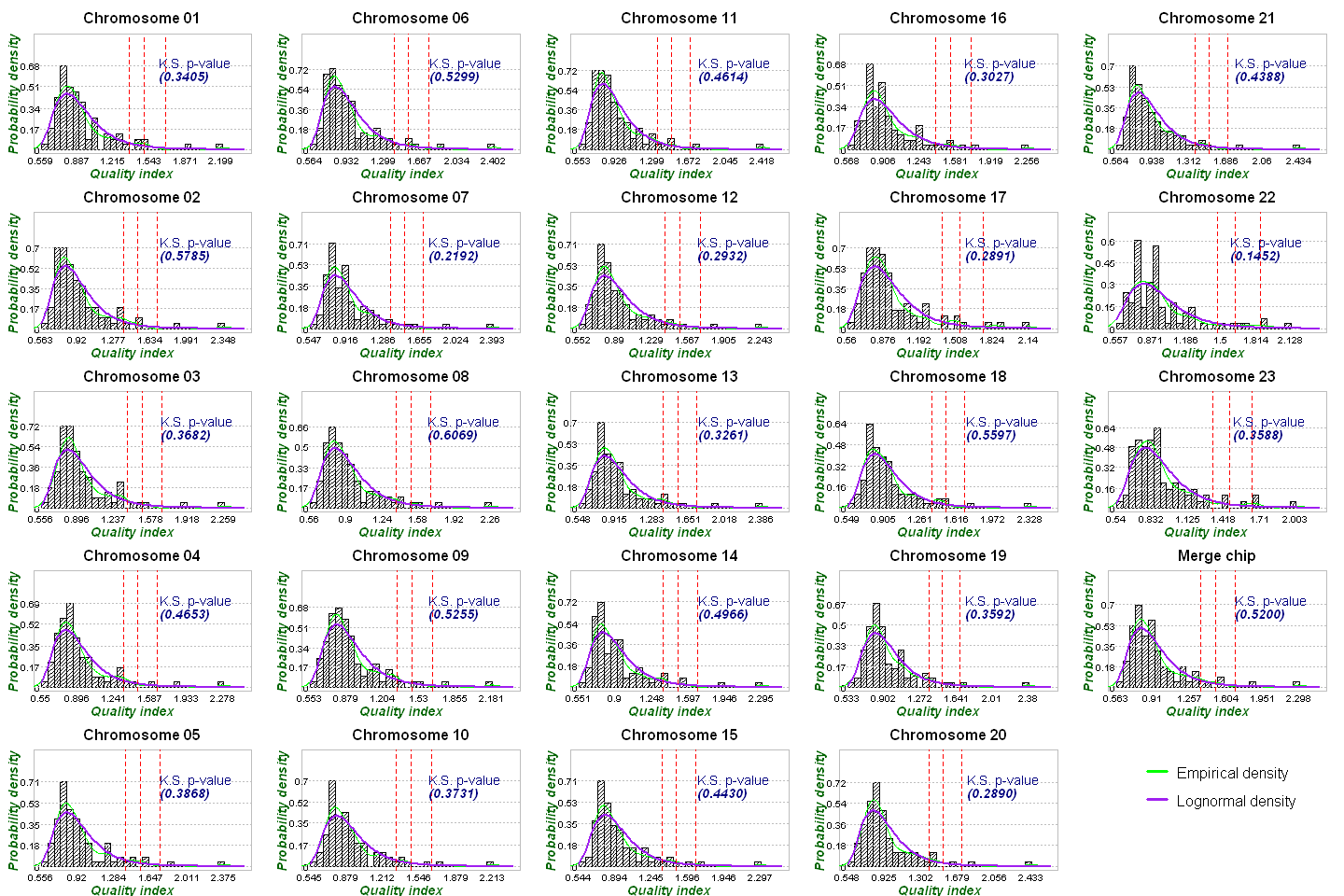


(A6)


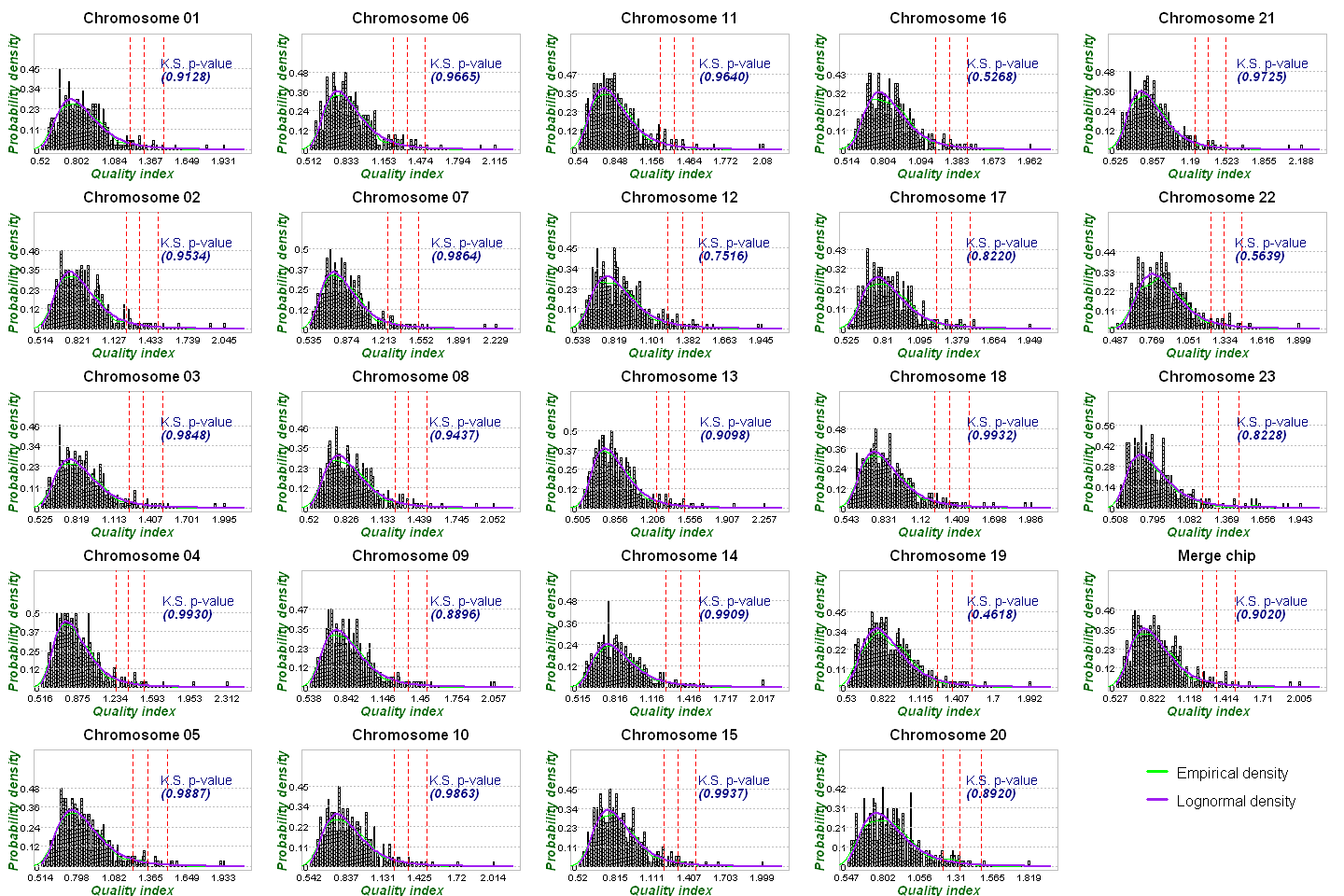


(A7)


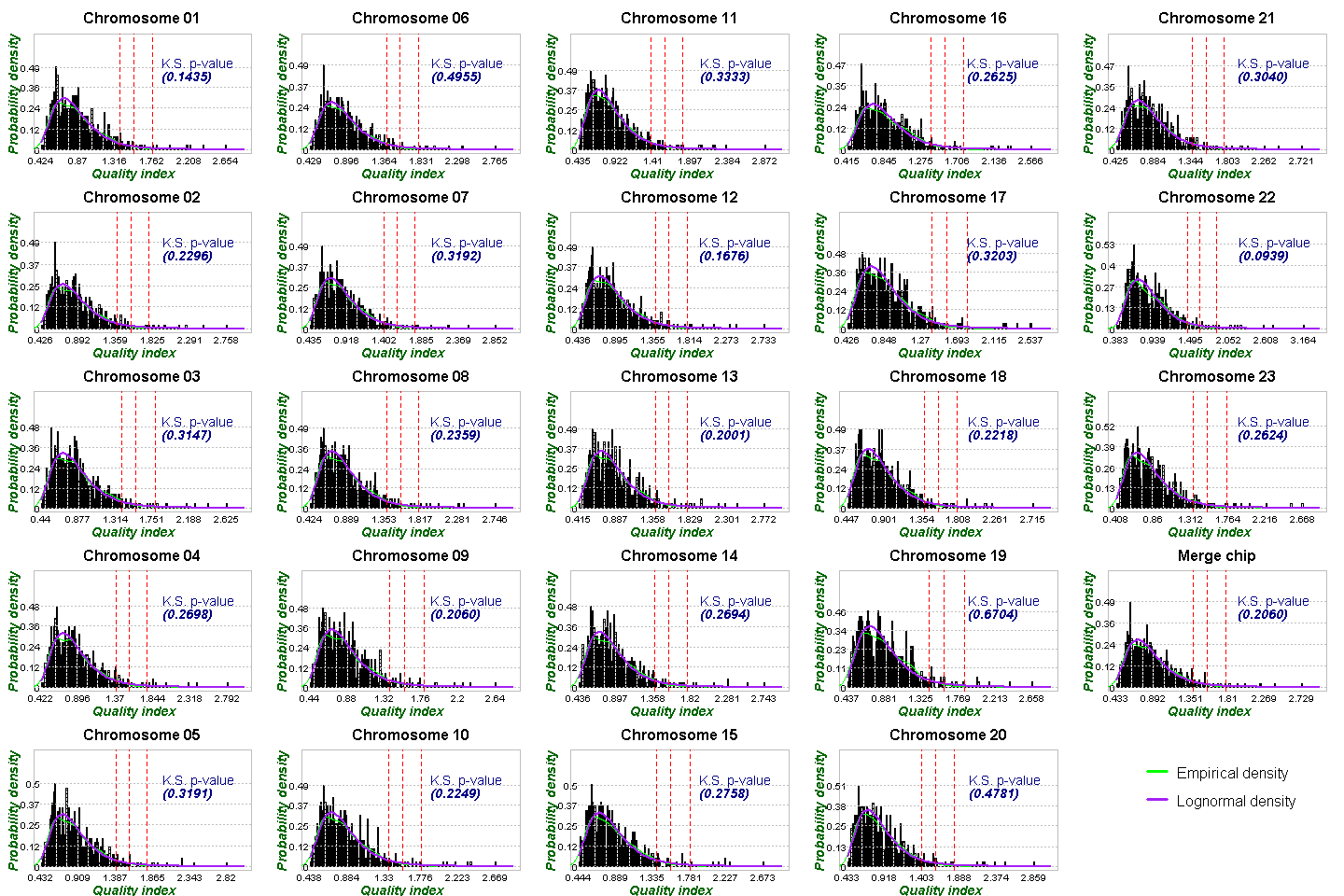


(B1)


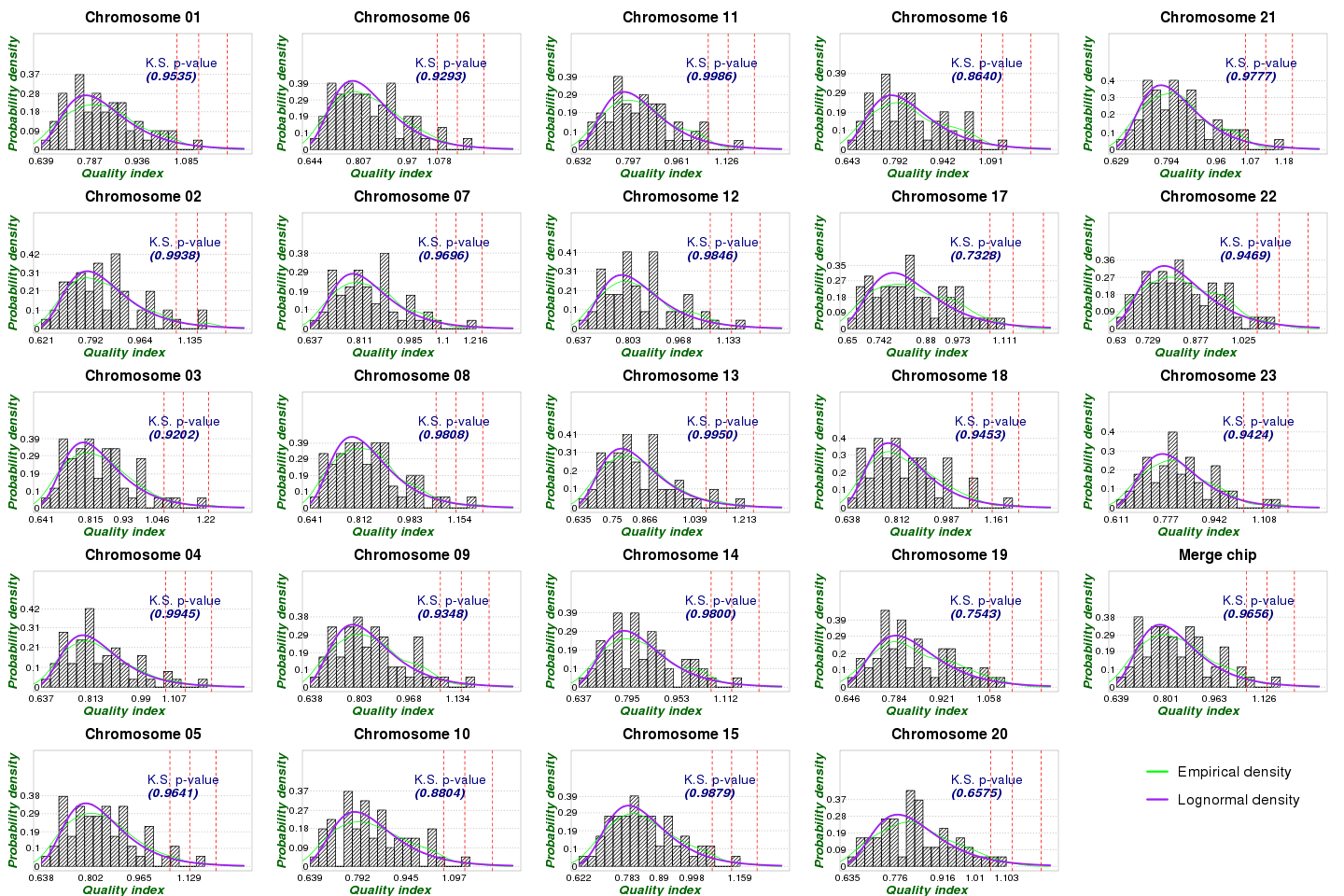


(B2)


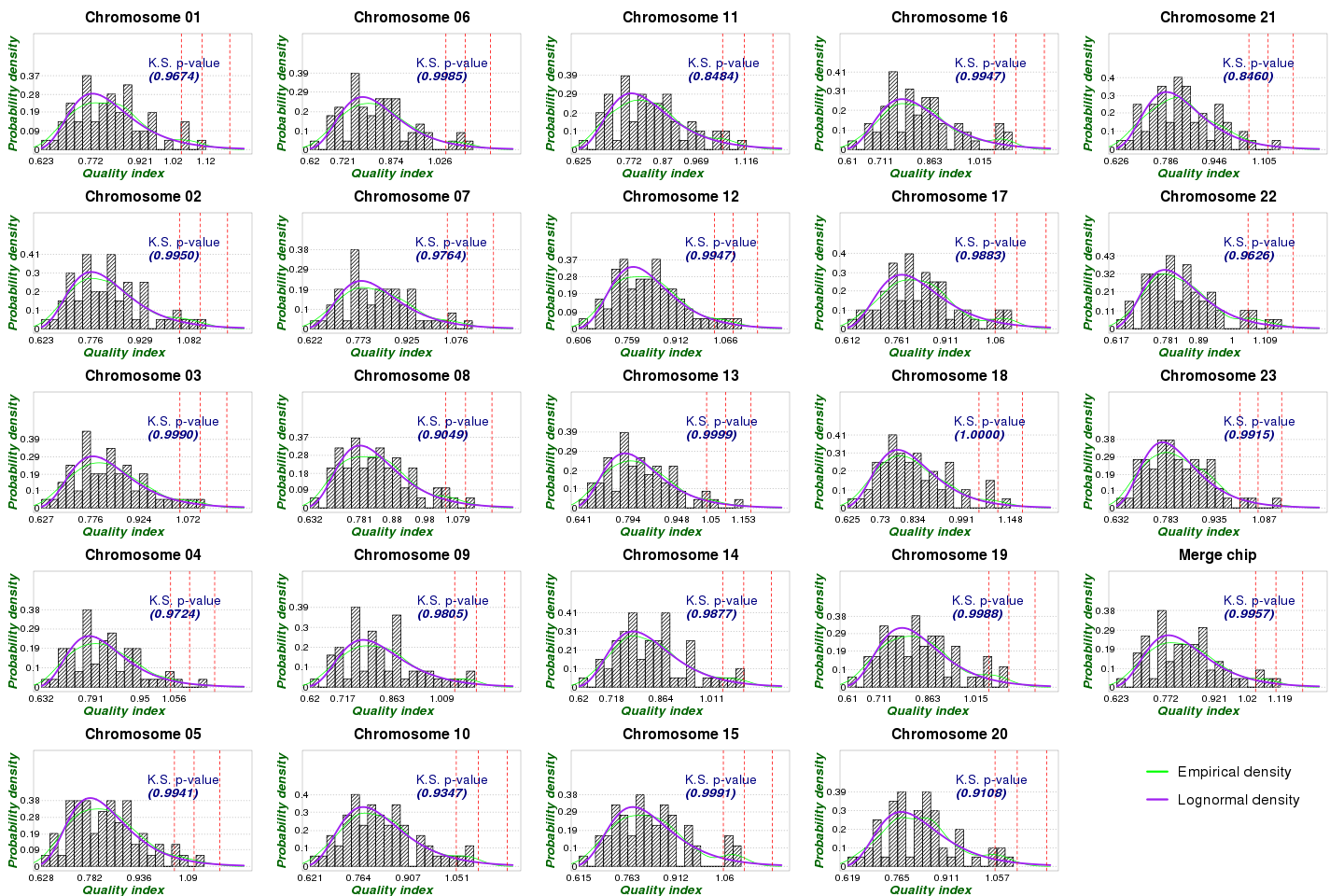


(B3)


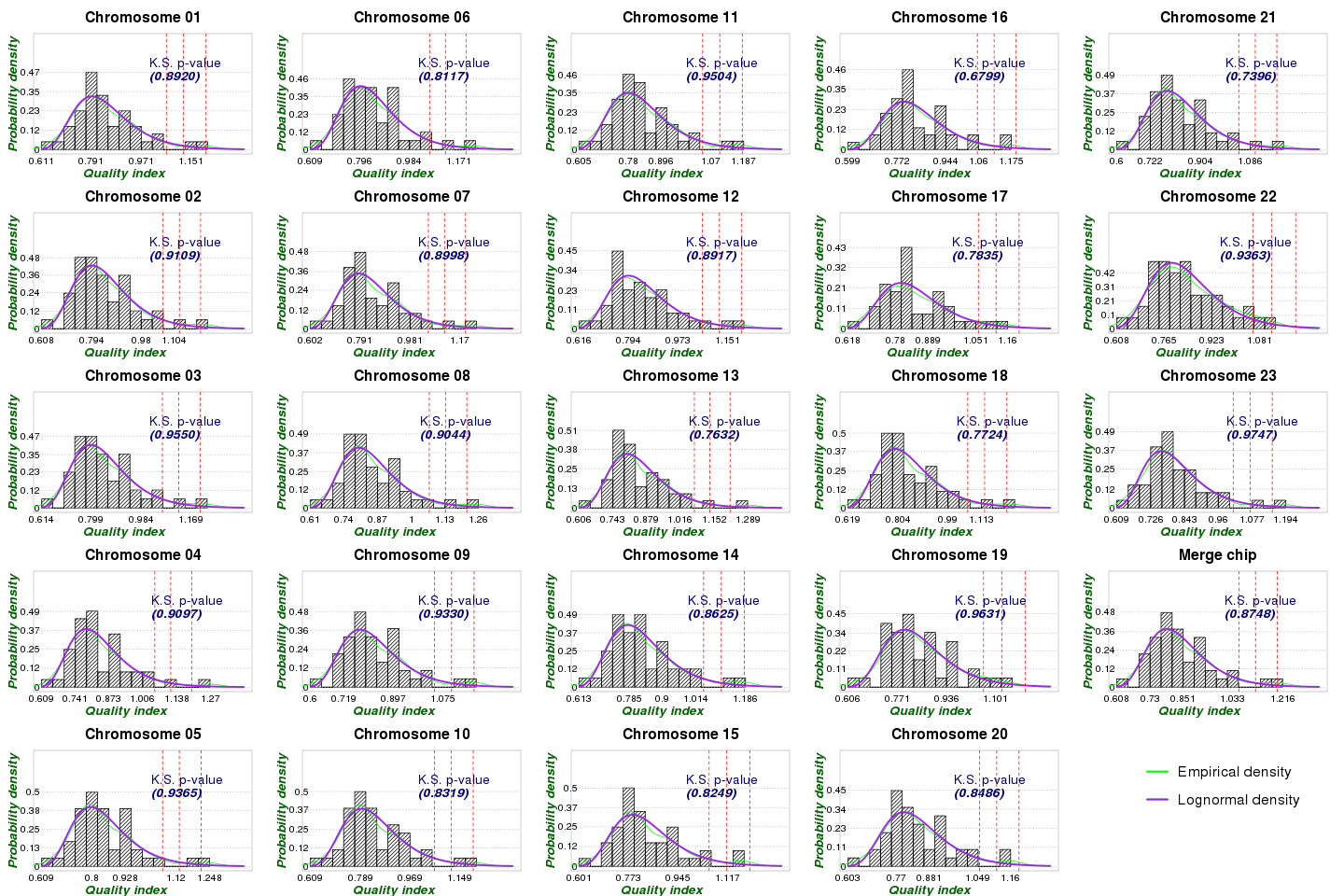


(B4)


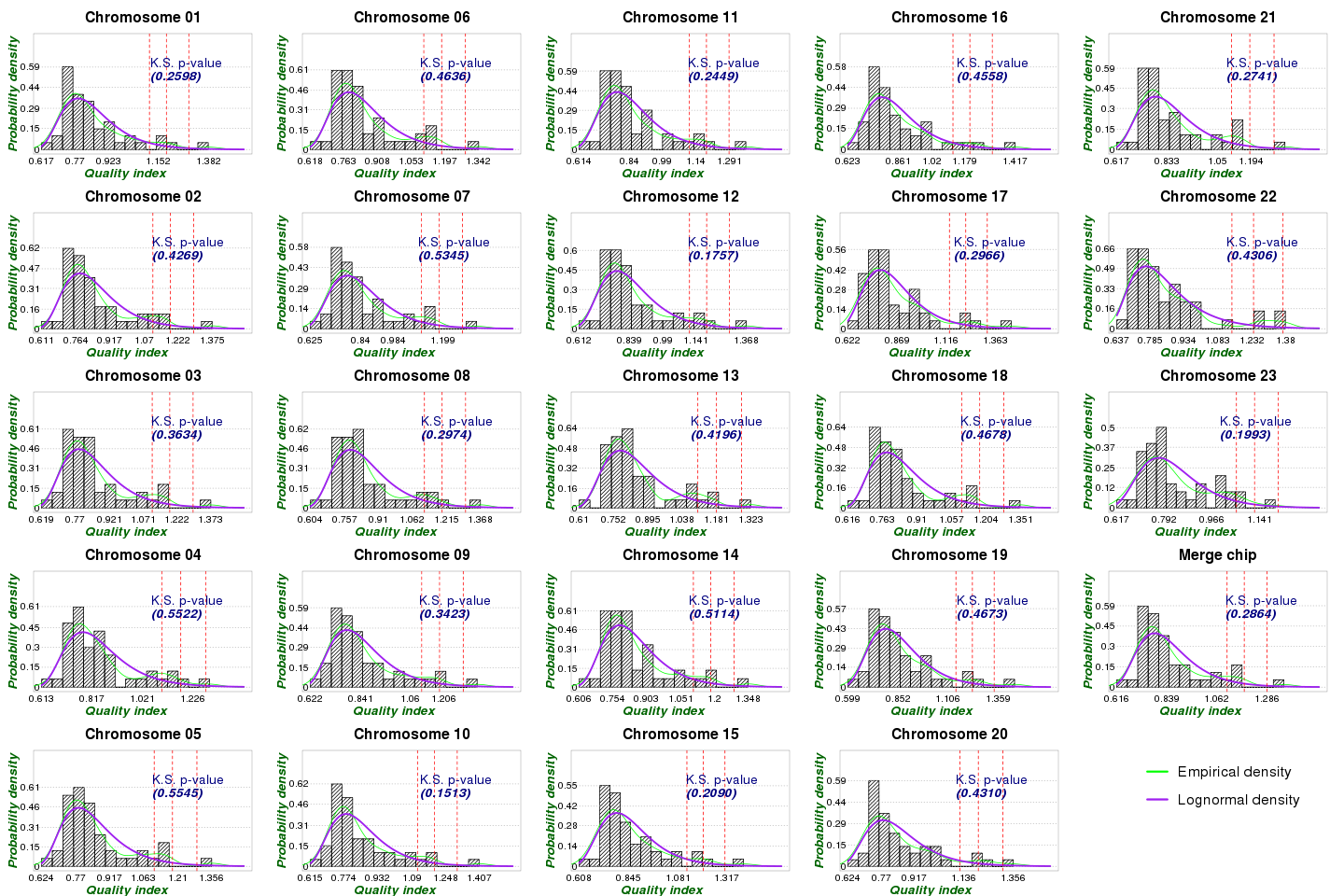


(B5)


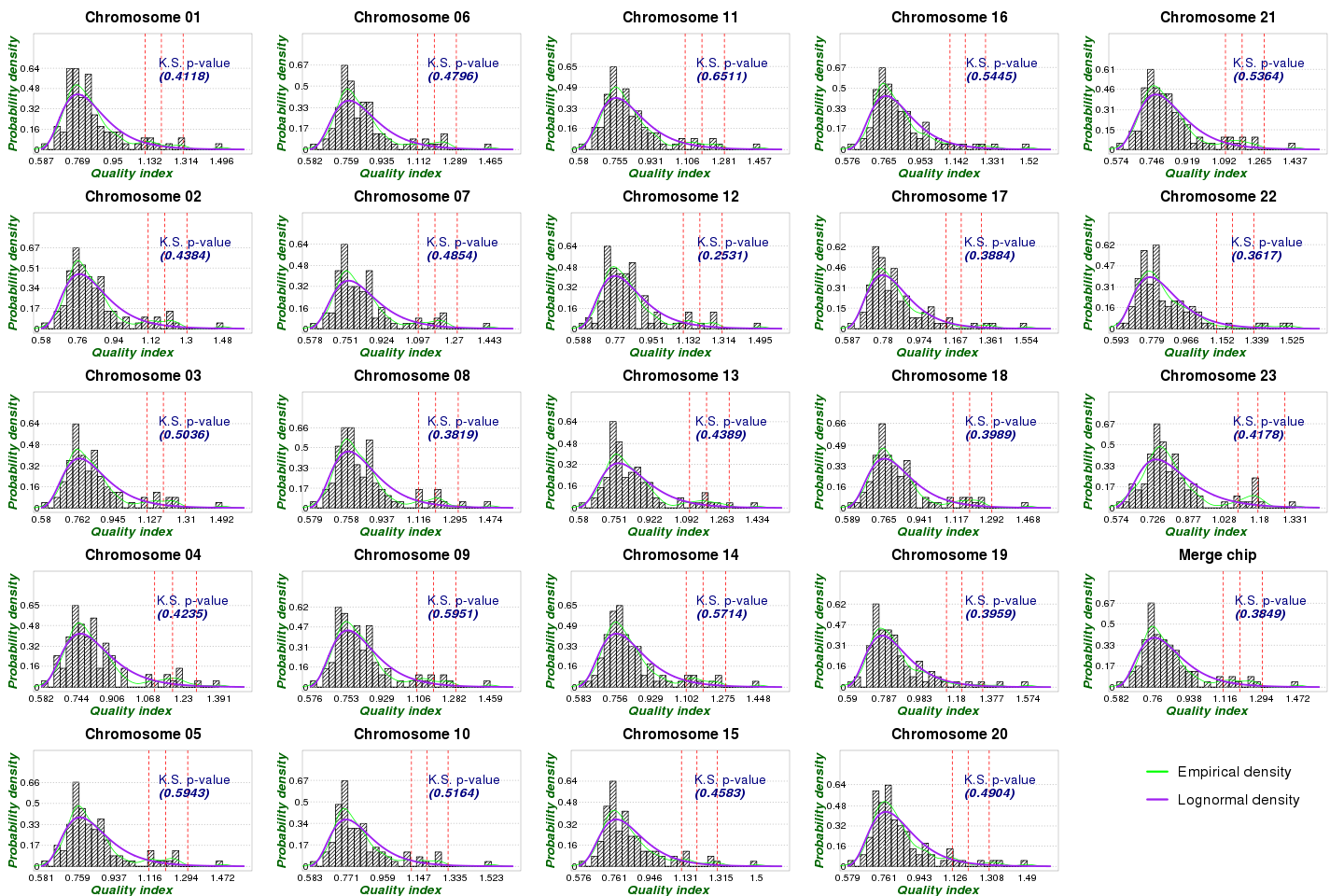


(B6)


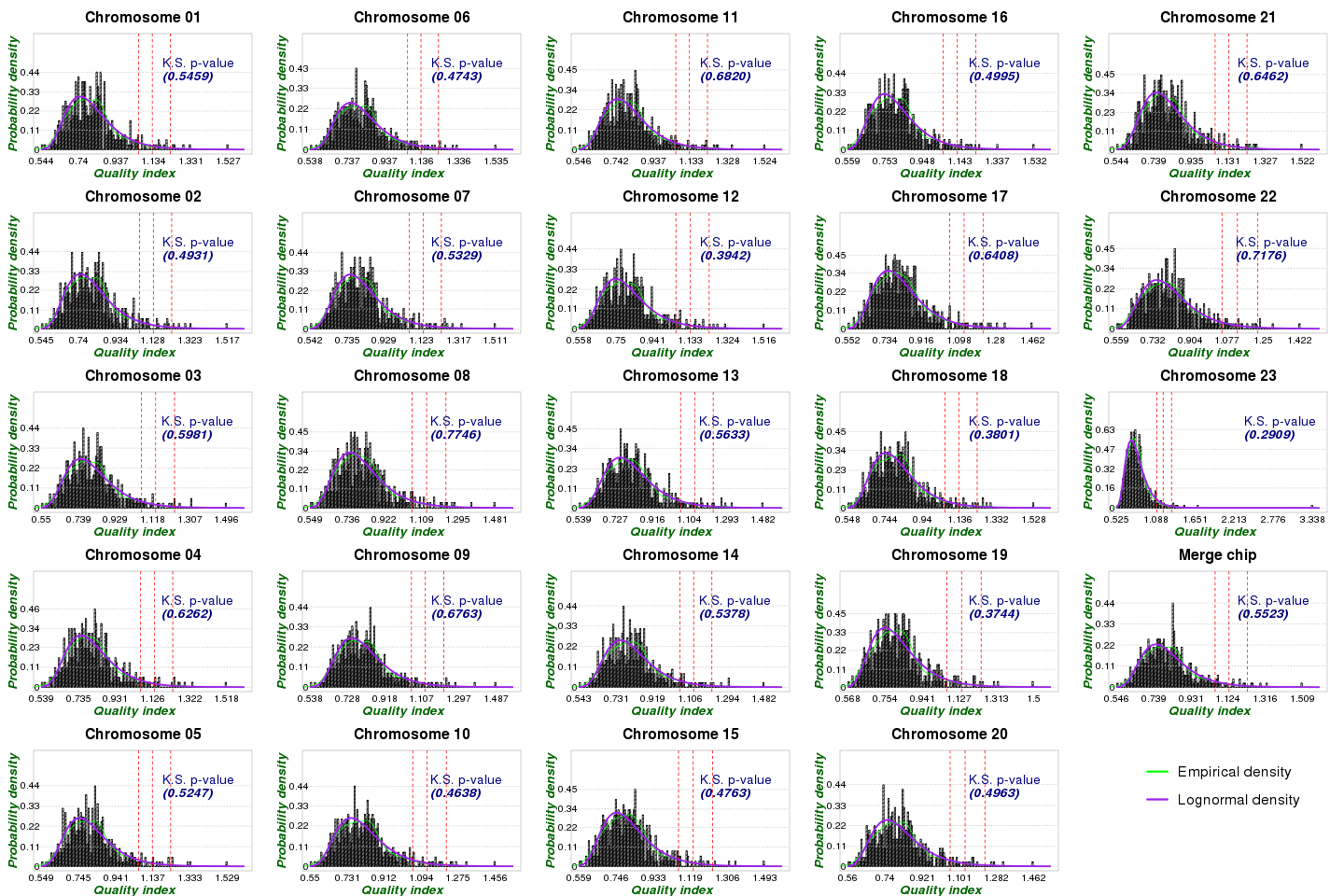


(B7)


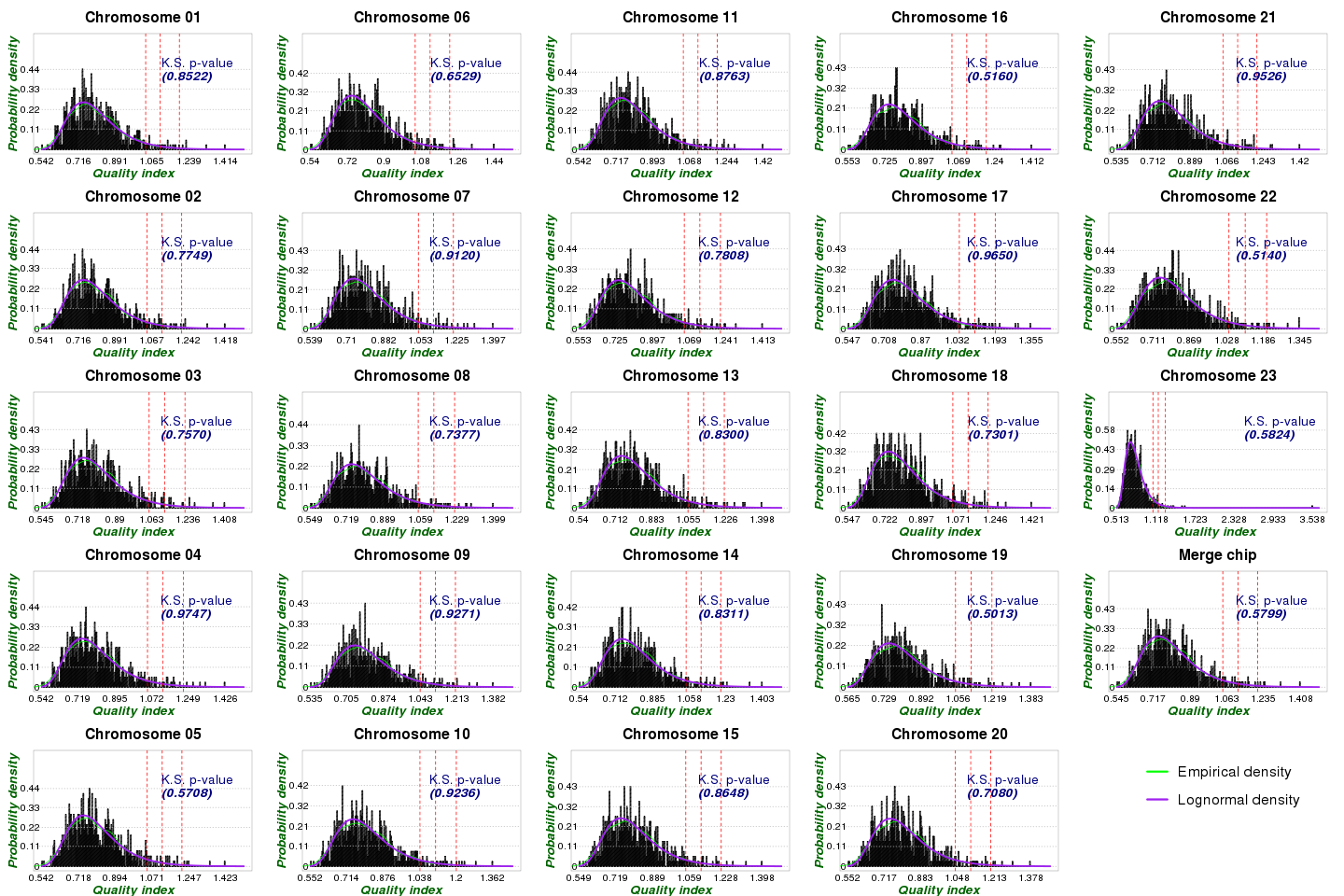

Supplement: Additional file 1 — Figure S1--Lognormal distribution of quality index based on the Affymetrix Human Mapping 100K and 500K Sets. Kolmogorov-Smirnov goodness-of-fit tests were used to examine lognormal distributions of the quality index Q2 for all study samples. Here, each figure consists of 24 panels. The first 23 panels show a distribution of the quality index for each chromosome, and the twenty-fourth panel presents a whole-genome distribution. In each panel, a histogram (gray bar), theoretical lognormal curve (purple line), and fitted curve (green line) for the quality index are shown, and the number shown in parentheses is the P-value of the Kolmogorov-Smirnov goodness-of-fit test. Three red dashed reference lines show the 95%, 97.5%, and 99% quantile. Samples with aneuploidy, amplification, or very long contiguous homozygous stretches were removed. For the Affymetrix Human Mapping 100K Set, we have (A1) 57 CEU founders, (A2) 58 YRI founders, (A3) 43 CHB samples, (A4) 43 JPT samples, (A5) 86 HapMap Asian samples (43 CHB and 43 JPT), (A6) 360 TWN samples, and (A7) 561 study samples (360 TWN samples and 201 HapMap samples). For the Affymetrix Human Mapping 500K Set, we have (B1) 55 CEU founders, (B2) 59 YRI founders, (B3) 43 CHB samples, (B4) 44 JPT samples, (B5) 87 HapMap Asian samples (43 CHB and 44 JPT), (B6) 442 TWN samples, and (B7) 643 study samples (442 TWN samples and 201 HapMap samples). [file 1471-2105-12-100-S1.DOC]
