# Supplementary material for: SAQC: SNP Array Quality Control
Source: BMC Bioinformatics. 2011 Apr 18;12:100. doi: 10.1186/1471-2105-12-100 (PMC3101186; doi:10.1186/1471-2105-12-100)
Supplement: Additional file 2 — Figure S2--Individual-level AF plots of four samples based on the Affymetrix Human Mapping 500K Set. AF plots of four samples: (A1) and (A2) are results of Nsp and Sty arrays for sample SC100011 (Sample 1); (B1) and (B2) are results of Nsp and Sty arrays for sample SC100854 (Sample 5); (C1) and (C2) are results of Nsp and Sty arrays for sample SC100444 (Sample 9) genotyped with expired SNP arrays; and (D1) and (D2) are results of Nsp and Sty arrays for pooled DNA samples (Sample 13). The panels display AFs for each of the 23 chromosomes. The horizontal axis is the physical position (unit = 1 Mb), and the vertical axis is the AF. Each SNP is denoted by a blue point, and the gap in each subplot represents the centromeric gap. The distribution of AFs was estimated using a smoothed density function and is shown as a pink curve. [file 1471-2105-12-100-S2.DOC]

**Figure S2.**—**Individual-level AF plots of four samples based on the Affymetrix Human Mapping 500K Set.** AF plots of four samples: (A1) and (A2) are results of Nsp and Sty arrays for Sample 1 genotyped with expired SNP arrays; and (B1) and (B2) are results of Nsp and Sty arrays for pooled DNA samples (Sample 5);

(C1) and (C2) are results of Nsp and Sty arrays for Sample 9; (D1) and (D2) are results of Nsp and Sty arrays for Sample 13. The panels display AFs for each of the 23 chromosomes. The horizontal axis is the physical position (unit = 1 Mb), and the vertical axis is the AF. Each SNP is denoted by a blue point, and the gap in each subplot represents the centromeric gap. The distribution of AFs was estimated using a smoothed density function and is shown as a pink curve.

**(A1)**

**
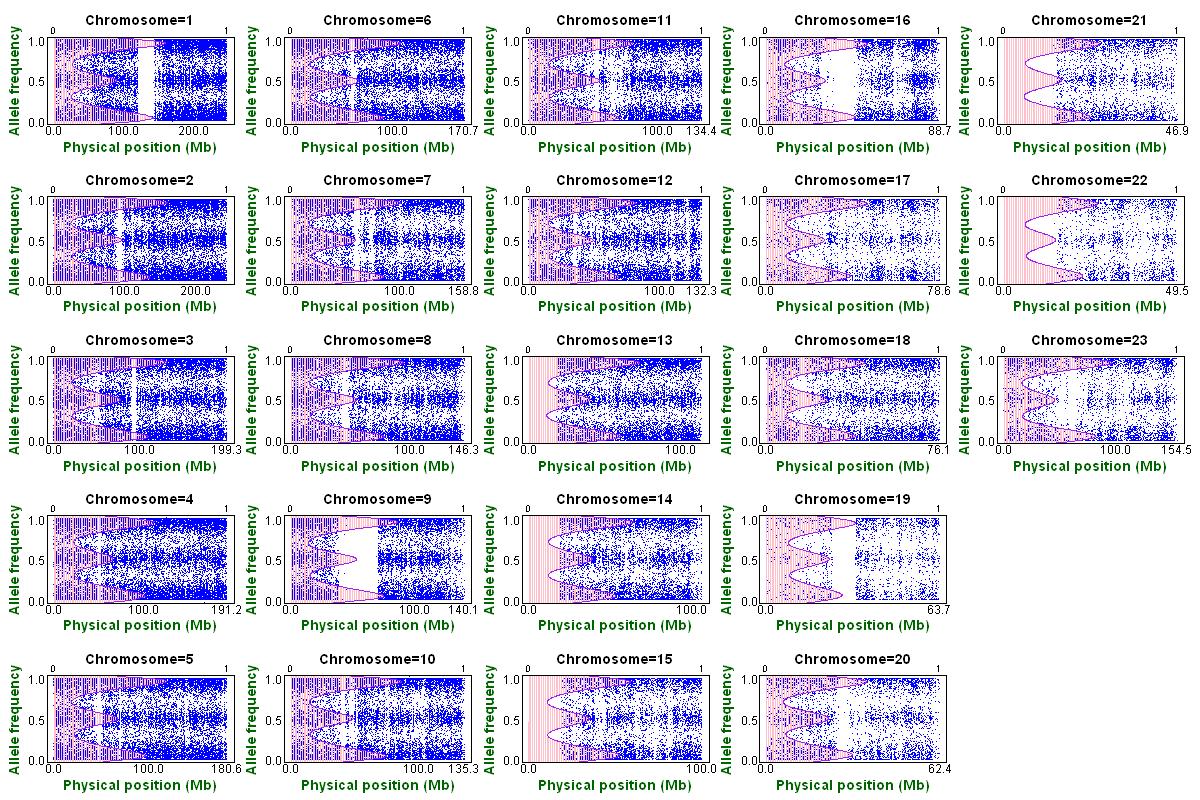
**

**(A2)**

**
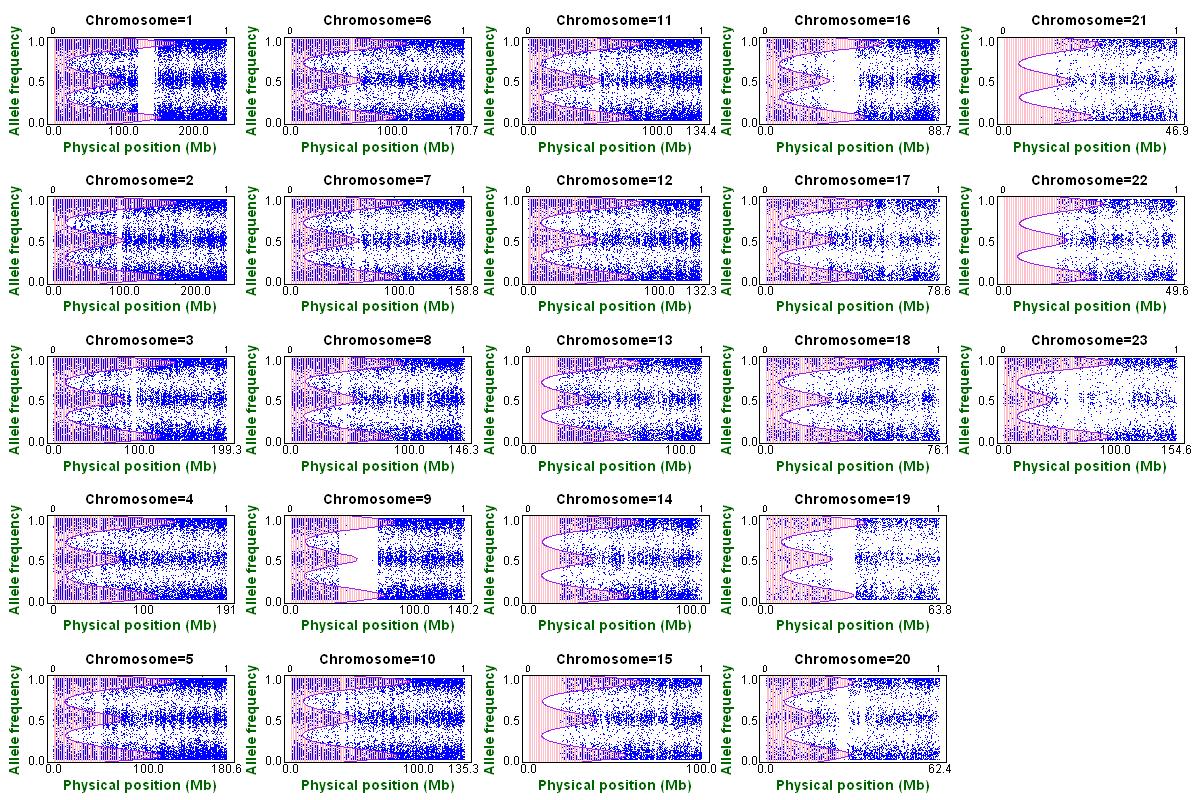
**

**(B1)**

**
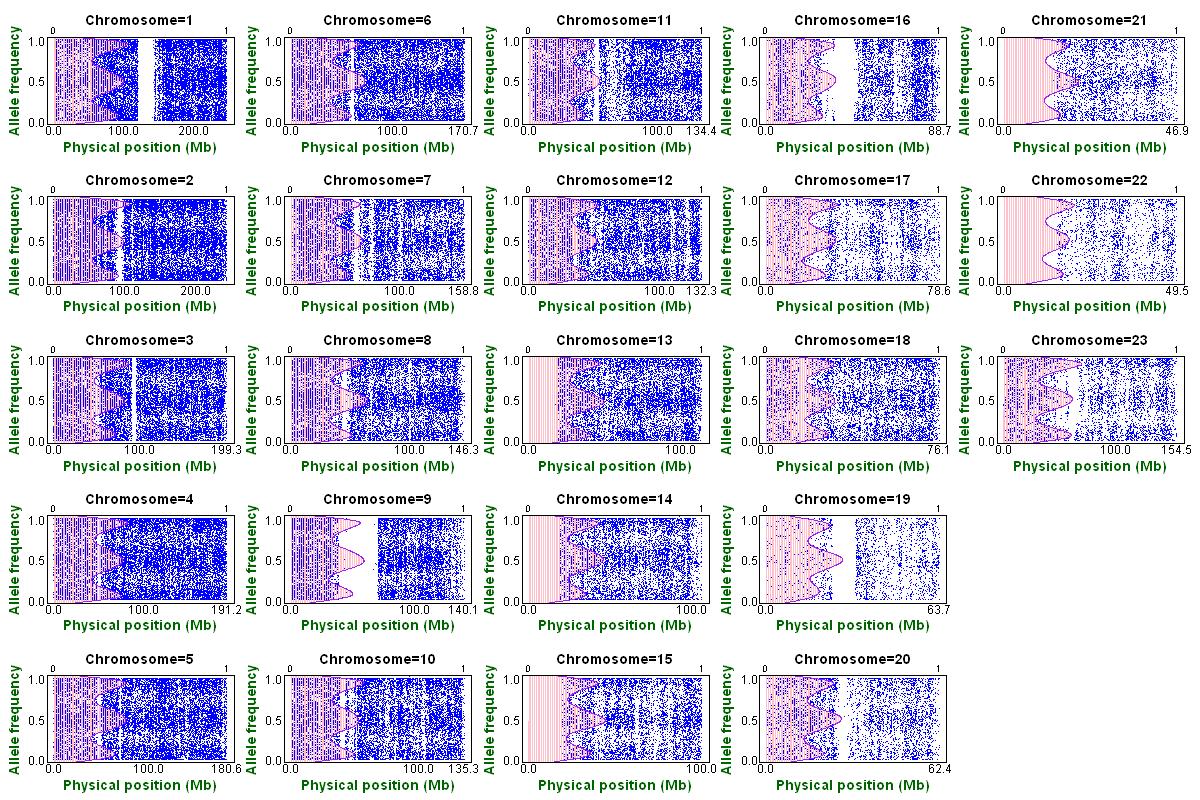
**

**(B2)**

**
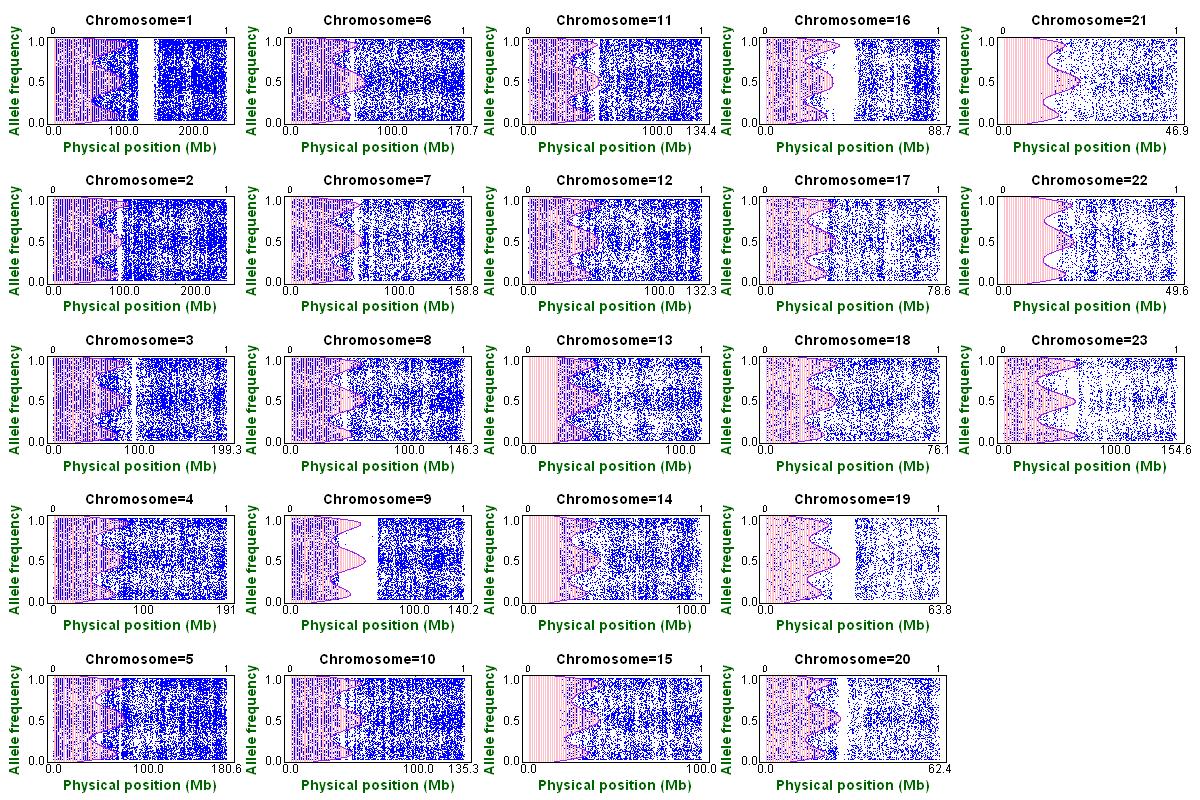
**

**(C1)**


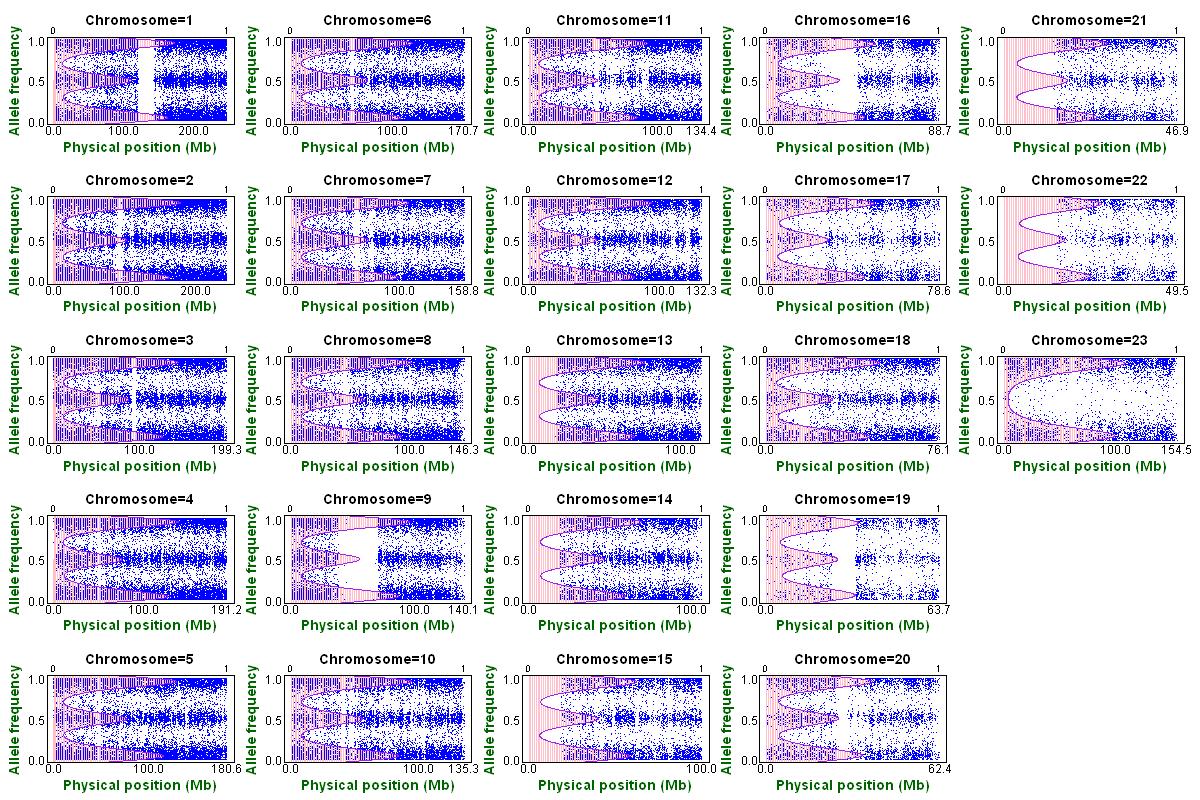


**(C2)**

**
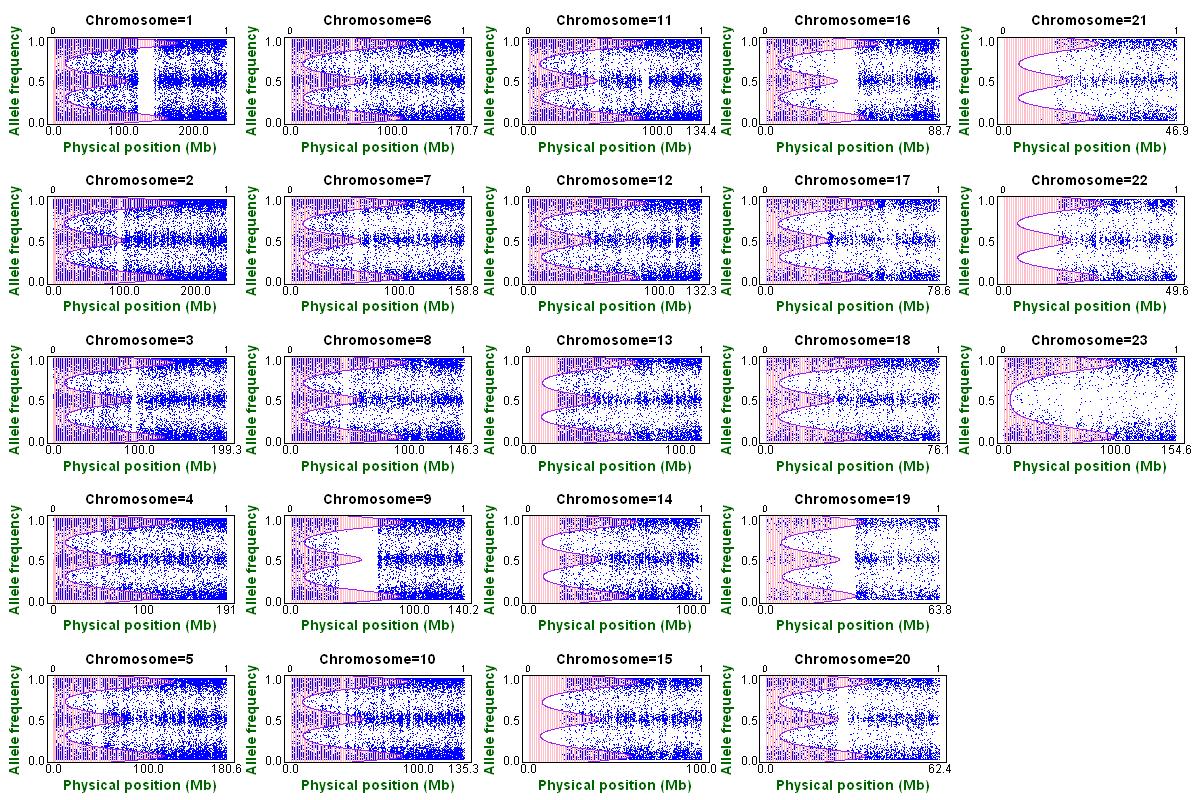
**

**(D1)**


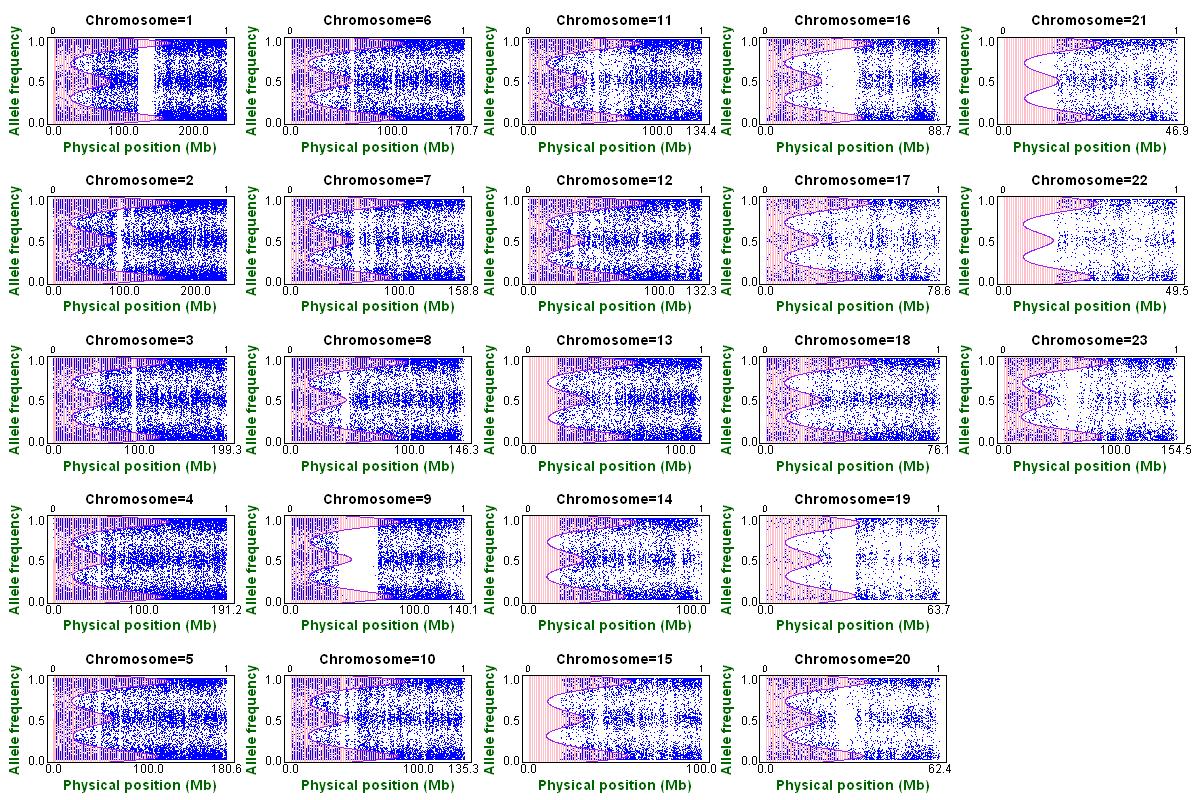


**(D2)**

**
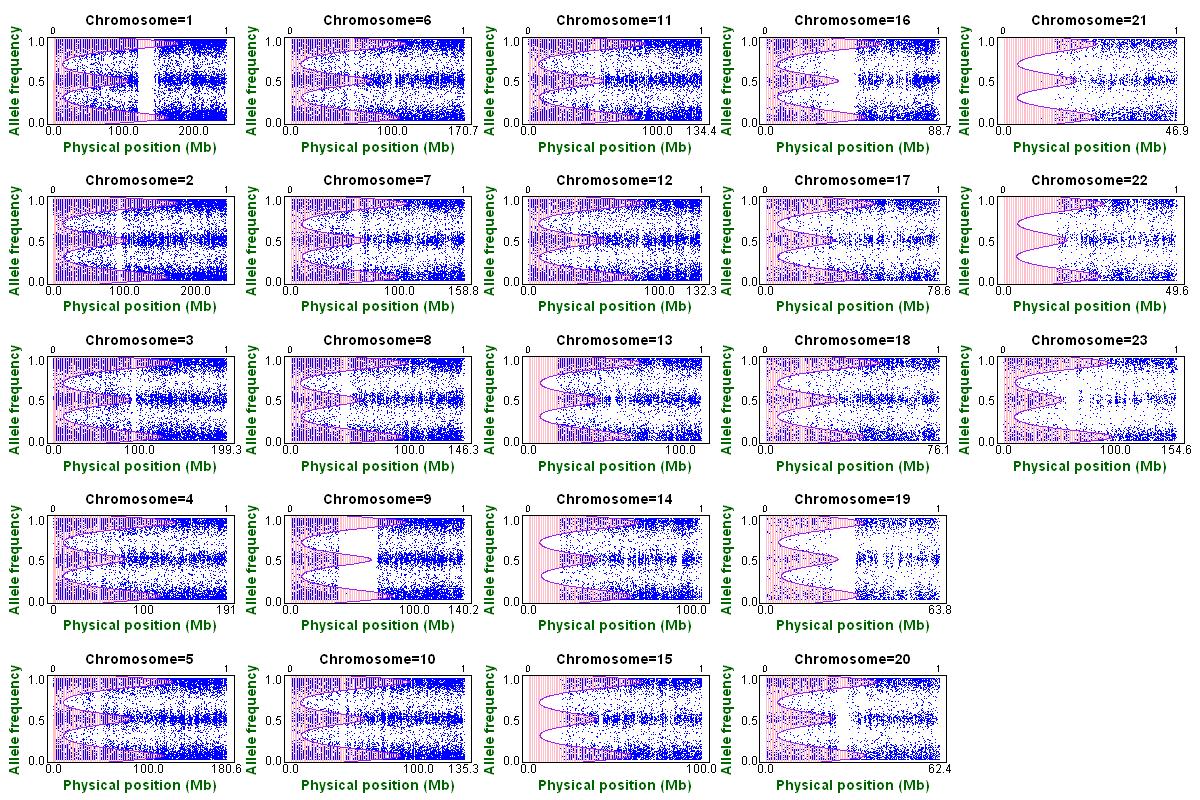
**
