# Supplementary material for: SAQC: SNP Array Quality Control
Source: BMC Bioinformatics. 2011 Apr 18;12:100. doi: 10.1186/1471-2105-12-100 (PMC3101186; doi:10.1186/1471-2105-12-100)

**Figure S4.**—**Two interactive plots provided by SAQC software.** (A) Interactive QI heatmap plot. (B) Interactive QI polygon plot.

(A)


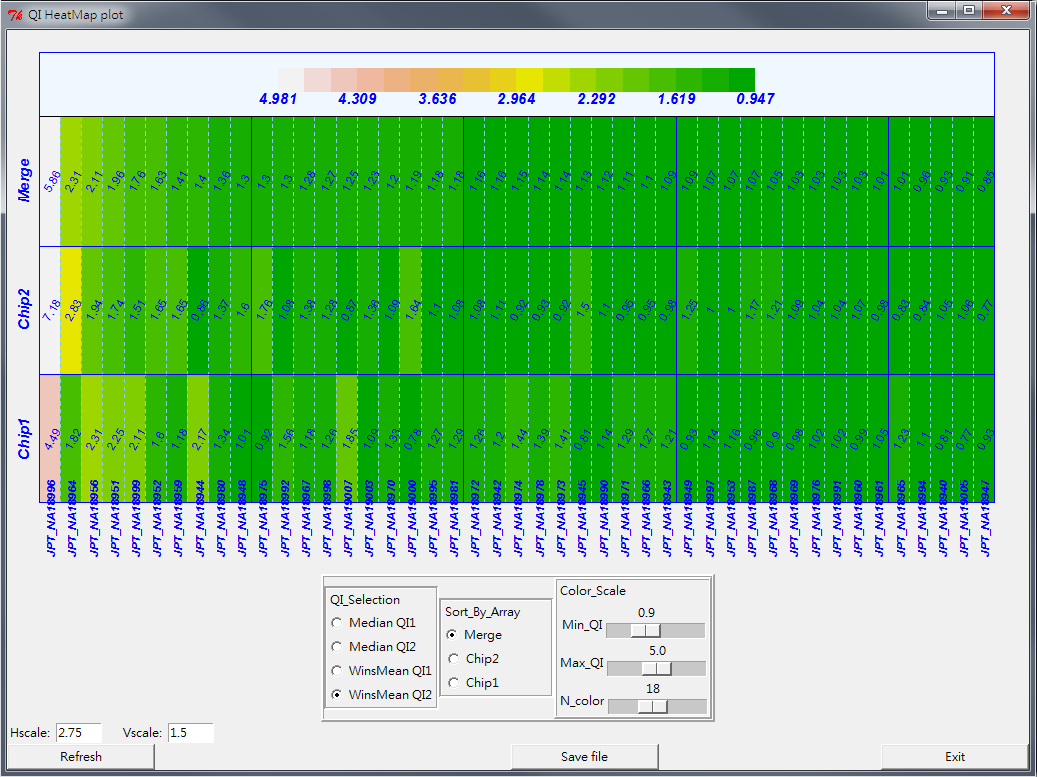


(B)


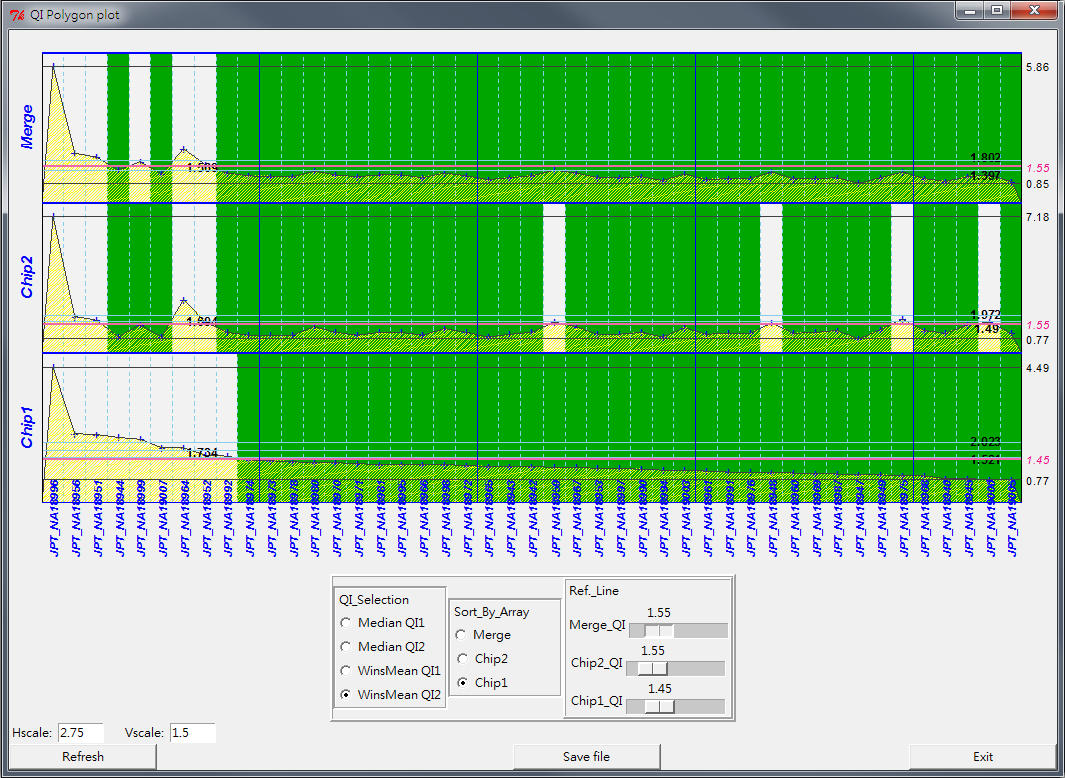

Supplement: Additional file 4 — Figure S4--Two interactive plots provided by SAQC software. (A) Interactive QI heatmap plot. (B) Interactive QI polygon plot. [file 1471-2105-12-100-S4.DOC]
