# Supplementary material for: SAQC: SNP Array Quality Control
Source: BMC Bioinformatics. 2011 Apr 18;12:100. doi: 10.1186/1471-2105-12-100 (PMC3101186; doi:10.1186/1471-2105-12-100)

**Figure S5.**—**Detection rates of median-based quality indices in the simulation study.** Averages and standard deviations of detection rates of the genotype-based index (QI1) and nearest-mean-based quality index (QI2) $\{Q_{1}(\rho),Q_{2}(\rho),\rho=95\%,97.5\%,99\%\}$ for a relative experimental error *r* of 0–60% with increments of 0.025. (A) HapMap Asian (CHB + JPT) population and Affymetrix 100K SNP array. (B) HapMap Asian (CHB + JPT) population and Affymetrix 500K SNP array. (C) The combined population (TWN + CHB + JPT + YRI + CEU) and Affymetrix 100K SNP array. (D) The combined population (TWN + CHB + JPT + YRI + CEU) and Affymetrix 500K SNP array.

(A)


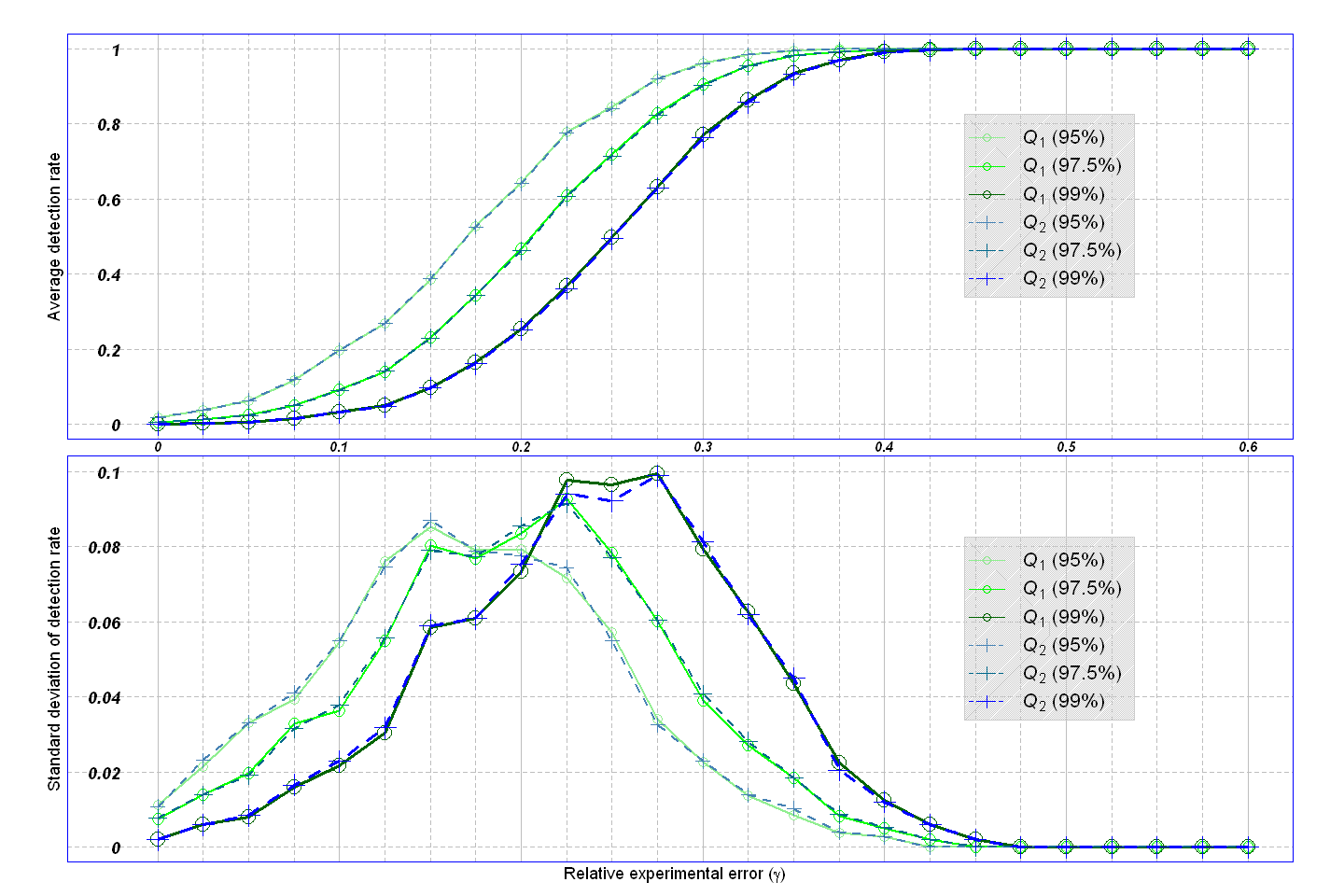


(B)


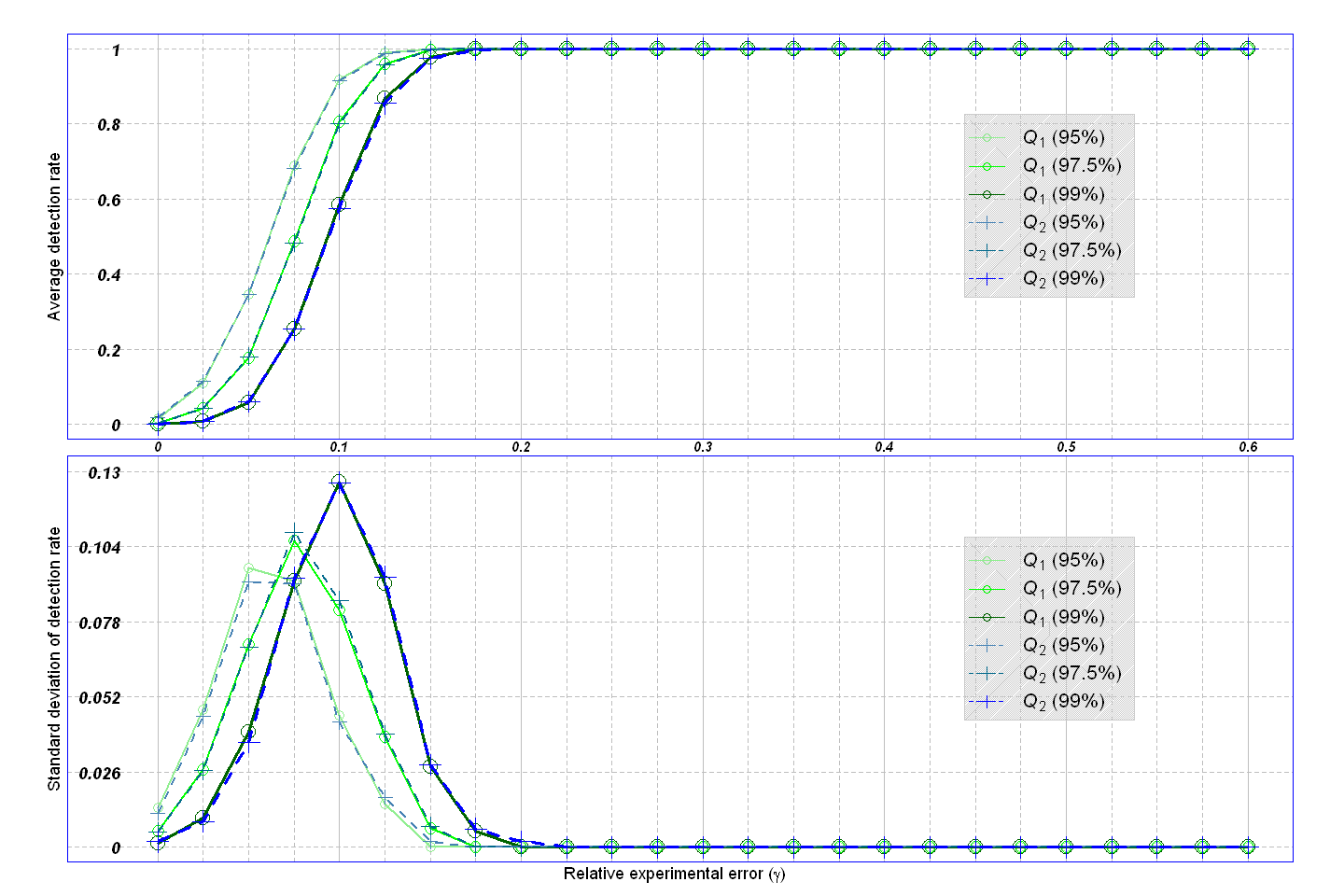


(C)


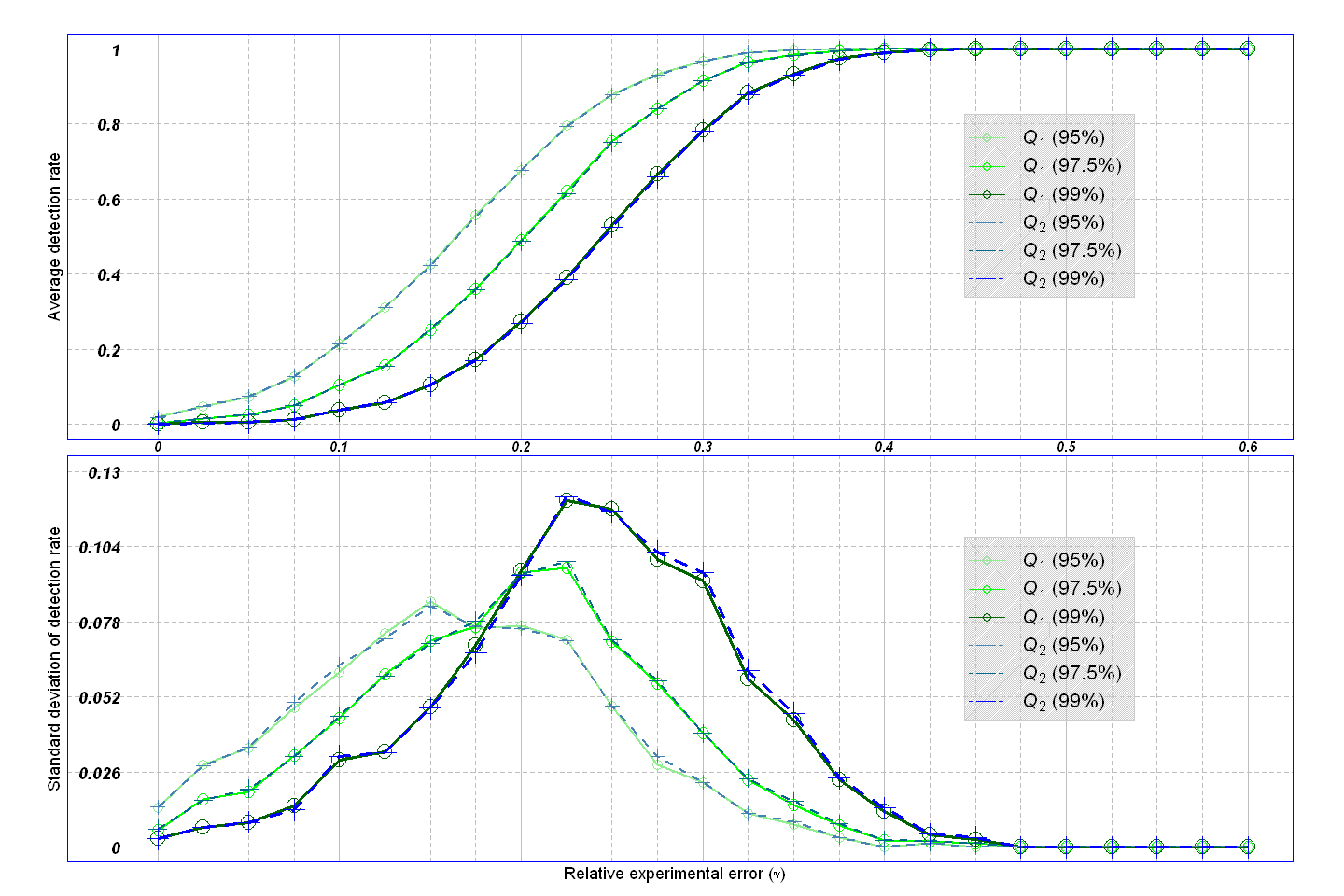


(D)


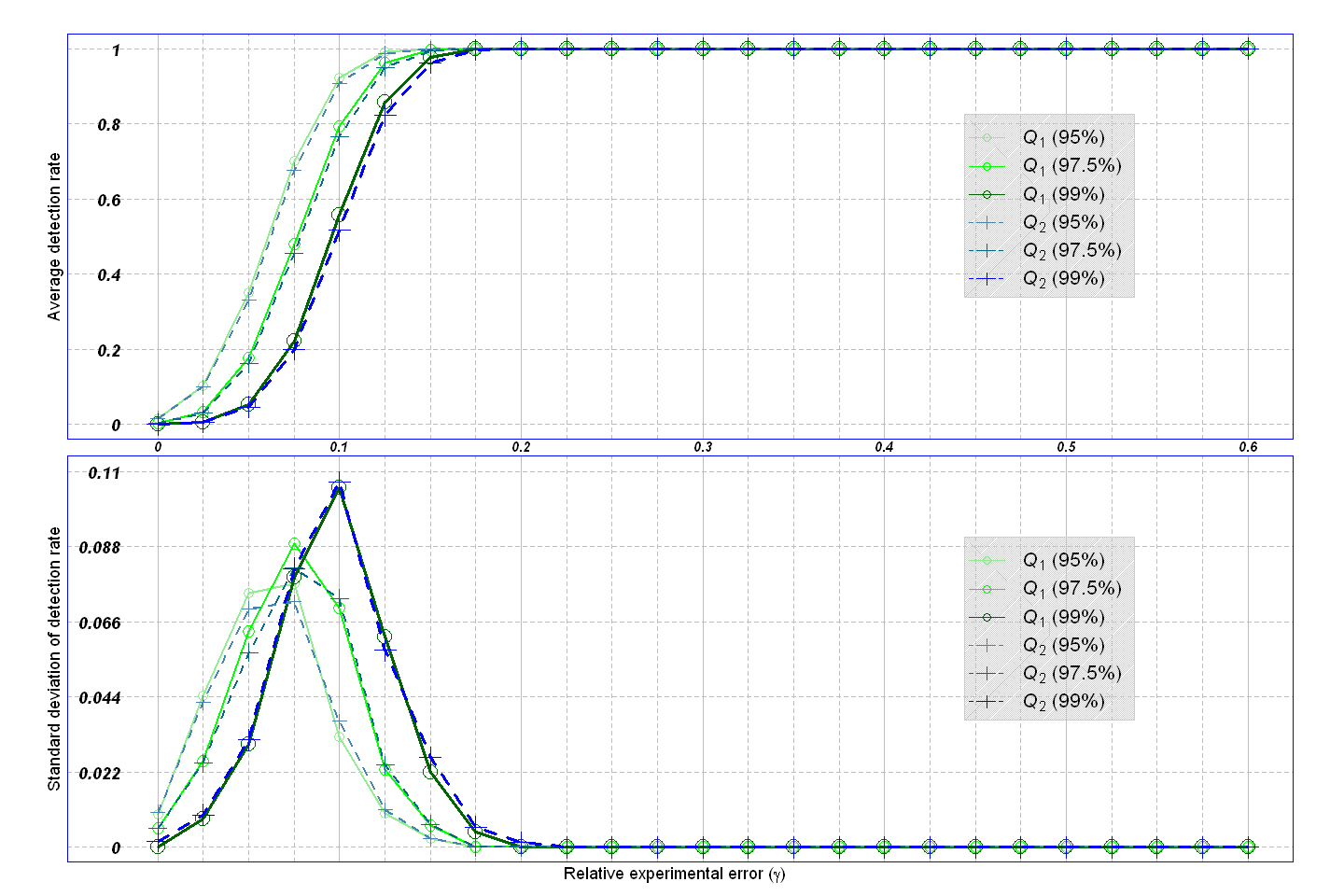

Supplement: Additional file 5 — Figure S5--Detection rates of median-based quality indices in the simulation study. Averages and standard deviations of detection rates of the genotype-based index (Q1) and nearest-mean-based quality index (Q2) {Q1(ρ), Q2(ρ), ρ = 95%, 97.5%, 99%} for a relative experimental error r of 0-60% with increments of 0.025. (A) HapMap Asian (CHB + JPT) population and Affymetrix 100K SNP array. (B) HapMap Asian (CHB + JPT) population and Affymetrix 500K SNP array. (C) The combined population (TWN + CHB + JPT + YRI + CEU) and Affymetrix 100K SNP array. (D) The combined population (TWN + CHB + JPT + YRI + CEU) and Affymetrix 500K SNP array. [file 1471-2105-12-100-S5.DOC]
