# Supplementary material for: SAQC: SNP Array Quality Control
Source: BMC Bioinformatics. 2011 Apr 18;12:100. doi: 10.1186/1471-2105-12-100 (PMC3101186; doi:10.1186/1471-2105-12-100)

**Figure S6.**—**Individual-level AF plot of a triploid cancer patient.** Individual-level AF data of a cancer patient were generated by a simulation procedure and then displayed in an AF plot. The panels display AFs for each of the 23 chromosomes. The horizontal axis indicates the physical position (unit = 1 Mb), and the vertical axis shows the AF. Each SNP is denoted by a blue point, and the gap in each subplot represents the centromeric gap. The distribution of AFs was estimated using a smoothed density function and is shown as a pink curve.


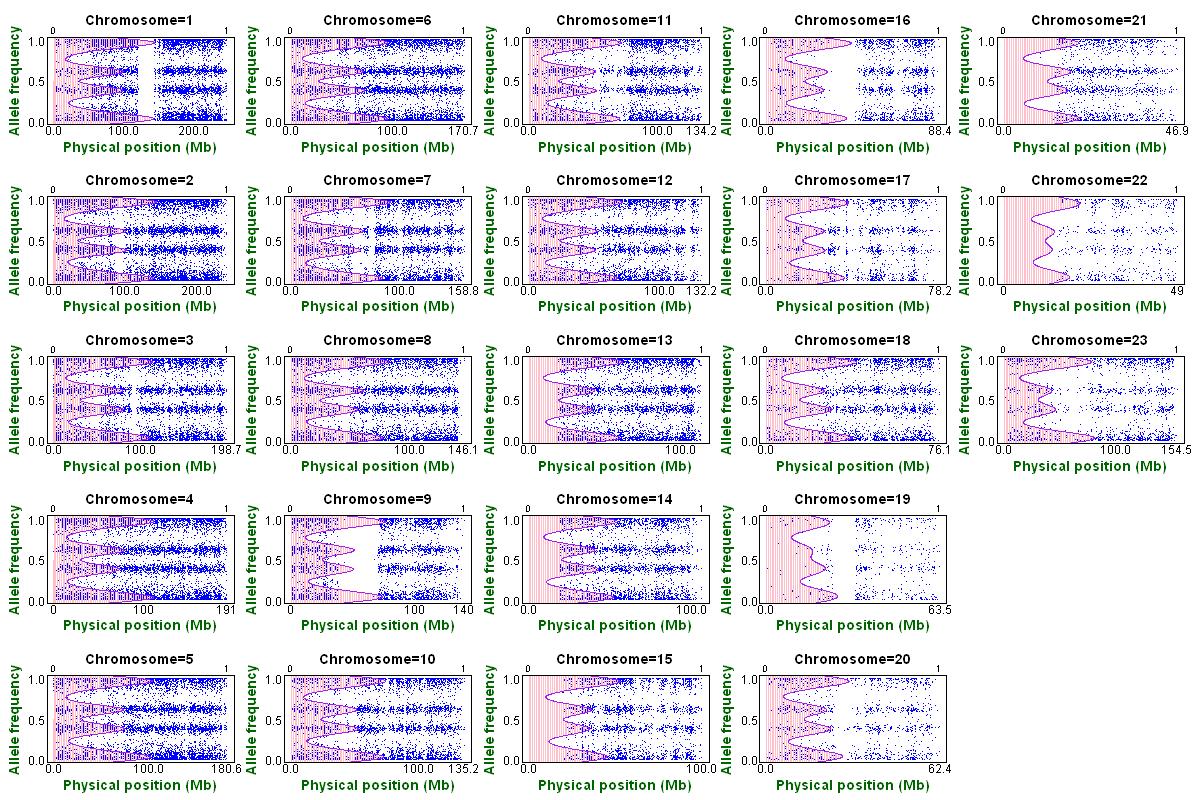

Supplement: Additional file 6 — Figure S6--Individual-level AF plot of a triploid cancer patient. Individual-level AF data of a cancer patient were generated by a simulation procedure and then displayed in an AF plot. The panels display AFs for each of the 23 chromosomes. The horizontal axis indicates the physical position (unit = 1 Mb), and the vertical axis shows the AF. Each SNP is denoted by a blue point, and the gap in each subplot represents the centromeric gap. The distribution of AFs was estimated using a smoothed density function and is shown as a pink curve. [file 1471-2105-12-100-S6.DOC]
